# Socioeconomic impacts on Andean adolescents’ growth: Variation between households, between communities and over time

**DOI:** 10.1093/emph/eoac033

**Published:** 2022-08-22

**Authors:** Mecca E Howe, Esperanza Caceres, Emily M Chester, Kathryn A Hicks, Thomas W McDade, Lynn Sikkink, Hilde Spielvogel, Jonathan Thornburg, Virginia J Vitzthum

**Affiliations:** Department of Anthropology, Indiana University, Bloomington, IN 47405, USA; Instituto Boliviano de Biología de Altura (IBBA), La Paz, Bolivia; Department of Anthropology, Indiana University, Bloomington, IN 47405, USA; Evolutionary Anthropology Laboratory, Indiana University, Bloomington, IN 47405, USA; Department of Anthropology, University of Memphis, Memphis, TN 38152, USA; Department of Anthropology, Northwestern University, Evanston, IL 60208, USA; Department of Anthropology, Western Colorado University, Gunnison, CO 81231, USA; Instituto Boliviano de Biología de Altura (IBBA), La Paz, Bolivia; Department of Astronomy & Center for Spacetime Symmetries, Indiana University, Bloomington, IN 47405, USA; Department of Anthropology, Indiana University, Bloomington, IN 47405, USA; Evolutionary Anthropology Laboratory, Indiana University, Bloomington, IN 47405, USA

**Keywords:** health disparities, adolescent growth, double burden of malnutrition, high-altitude adaptation, children's growth references, secular trend

## Abstract

**Background/Objectives:**

We evaluated potential socioeconomic contributors to variation in Andean adolescents’ growth between households within a peri-urban community undergoing rapid demographic and economic change, between different community types (rural, peri-urban, urban) and over time. Because growth monitoring is widely used for assessing community needs and progress, we compared the prevalences of stunting, underweight, and overweight estimated by three different growth references.

**Methods:**

Anthropometrics of 101 El Alto, Bolivia, adolescents (Alteños), 11.0–14.9 years old in 2003, were compared between households (economic status assessed by parental occupations); to one urban and two rural samples collected in 1983/1998/1977, respectively; and to the WHO growth reference, a representative sample of Bolivian children (MESA), and a region-wide sample of high-altitude Peruvian children (Puno).

**Results:**

Female Alteños’ growth was positively associated with household and maternal income indices. Alteños’ height averaged ∼0.8SD/∼0.6SD/∼2SDs greater than adolescents’ height in urban and rural communities measured in 1983/1998/1977, respectively. Overweight prevalence was comparable to the WHO, and lower than MESA and Puno, references. Stunting was 8.5/2.5/0.5 times WHO/MESA/Puno samples, respectively.

**Conclusions/Implications:**

Both peri-urban conditions and temporal trends contributed to gains in Alteños’ growth. Rural out-migration can alleviate migrants’ poverty, partly because of more diverse economic options in urbanized communities, especially for women. Nonetheless, Alteños averaged below WHO and MESA height and weight medians. Evolved biological adaptations to environmental challenges, and the consequent variability in growth trajectories, favor using multiple growth references. Growth monitoring should be informed by community- and household-level studies to detect and understand local factors causing or alleviating health disparities.

## 1. INTRODUCTION

Like many regions across the globe [1], climate change in the Bolivian altiplano has rendered rural economic strategies less tenable and driven individuals and families to migrate to urbanized areas in search of employment [2, 3]. Numerous economic, social and health effects, both beneficial and detrimental, are associated with this migration to urban centers and adjacent peri-urban communities. Depending on the specific conditions of natal and destination communities, children may be particularly vulnerable, or may especially benefit, from these population shifts [3–5]. In more urbanized settings, greater reliance on processed foods and less physical activity may raise the risk for stunting in early life and obesity in adulthood, a pattern often referred to as the ‘double burden of malnutrition’ [6]. On the other hand, these same conditions may provide immediate relief from arduous labor and seasonal food shortages, common in some agricultural regions, thereby reducing the risk of childhood undernutrition and stunting.

For several decades, El Alto has been the major peri-urban destination for rural migrants in highland Bolivia and is still expanding in both population and economic diversity (see ‘Supplementary Data: Study Setting’ for details). These developments afforded a novel opportunity to evaluate the impacts of household, community and regional temporal trends in socioeconomic conditions on Andean adolescent’s growth. A better grasp of which factors, operating at the individual household and/or at more aggregated levels, influence health outcomes can potentially inform the development of more effective intervention programs. We addressed these questions with data collected from a sample of El Alto adolescents (Alteños) in 2003, by which time El Alto had grown to be the third largest Bolivian city but with some two-thirds of its population not having their basic needs met [3, 7, 8].

From its inception, a foundational purpose of our project in El Alto was to contribute to the small body of data on adolescent growth and development in middle- and low-income countries. Progress on this front has been slow. Many large surveys (e.g. the Demographic Health Surveys) that include subadults typically focus only on infants and young children, a reflection of higher mortality at these younger ages and a recognition that early development has long-term impacts on health through adulthood.

Nonetheless, adolescence is the only time besides infancy when the growth rate accelerates, potentially affording an opportunity for catch-up growth that may compensate for growth faltering from harshness experienced in earlier life [9, 10]. In addition, the tempo and magnitude of adolescent weight gain may be a bellwether for adult obesity, itself a risk factor for several chronic diseases. Because adolescence is the final growth stage before adulthood, a better understanding of the dynamic interaction between evolutionary processes and socioeconomic contexts that generates variation in adolescent maturation within and between populations is sorely needed.

### 1.1 Study goal 1: Background and hypotheses

Our primary goal was to evaluate hypothesized socioeconomic determinants of El Alto adolescents’ growth. Specifically, we examined (i) the effects of economic variability across El Alto households, (ii) the effects of different types of communities (rural, urban and peri-urban) and (iii) the effects of regional temporal (secular) economic trends.

#### 1.1.1 The effects of economic variability across El Alto households

For some rural out-migrants, the wider range of income-generating options in peri-urban settings may provide the possibility of improving household income. But for other migrants, peri-urban employment may be no more than an erratic succession of low-paying jobs with little economic security. Nonetheless, in contrast to the few means rural women have for earning income, the potentially greater economic opportunities for women in a peri-urban economy may favorably tip the economic scales for many migrant households.

These various options to improve household income are likely to differentially impact child growth across El Alto households. We predicted a positive association between the anthropometrics of Alteño adolescents and their family’s socioeconomic status (SES), operationalized by (i) household income as indexed by parental occupations (i.e. paternal income index (PII) and maternal income index (MII)), and by (ii) parents’ relative use of Spanish versus an indigenous language (the use of Spanish was found to be positively associated with children’s anthropometrics in a rural Bolivian community [11]).

#### 1.1.2 The effects of different community types

Community types differ in services, infrastructure, density, social dynamics and many other factors, most of which may impact children’s growth. Therefore, we evaluated whether adolescent growth patterns in El Alto differed from those of adolescents in a central urban and two rural communities (described in Section 2.2), all at comparable altitude.

Compared to the rural communities, we expected El Alto’s better public services (e.g. sanitation, water, healthcare) and greater diversity of income-generating opportunities for adults would lead to improved physical growth in their children. Therefore, we predicted that our study sample of Alteño adolescents would, on average and adjusted for age and sex, be taller and have higher adiposity than their counterparts in rural communities.

In their nationwide analysis, O’Hare and Rivas [3] addressed the question of whether rural to urban migration in Bolivia had alleviated poverty levels or merely shifted its location. Based on aggregate (municipal and department) economic indicators, they concluded that migration was associated with alleviating poverty, largely because cities were better able than rural areas to supply basic services. Our comparative analyses of El Alto to rural communities are thus also a test of O’Hare and Rivas’ conclusion; if correct, El Alto adolescents’ growth should be improved compared to the rural communities.

We also compared the Alteño sample to a 1983 sample of La Paz school children from lower-middle income urban neighborhoods [12]. It is not clear *a priori* which of these two samples will be taller or have higher adiposity, or whether they may be similar. Despite better municipal services in La Paz, its higher costs, congestion, pollution, crowded housing, greater distance from natal communities and loss of traditional support networks pose new threats to migrant health and well-being. Hence, El Alto in 2003 may have been a more advantageous environment for migrants than La Paz.

#### 1.1.3 The effects of temporal (secular) trends

Because the studies in the communities we compared were conducted at different times, we also estimated the relative contributions of temporal/secular trends versus community type to any differences in adolescent growth observed between the compared community samples. Positive or negative secular trends in physical growth are generally attributable to changes in living conditions, broadly speaking [13]. The strength and consistency of this association are such that Pawson and Huicho [14] suggested temporal changes in growth in high-altitude communities ‘may be a reliable yardstick for assessing long-term changes in socioeconomic conditions…’. In the 1990s, Bolivia undertook infrastructure development initiatives for new water and sewage connections, and new electricity and telephone services, in the poorest neighborhoods of El Alto and La Paz [15]. Because of these developments, the peri-urban Alteño sample may compare favorably to the earlier urban Paceño sample.

### 1.2 Study goal 2: Background and hypotheses

Our second goal was to compare the different estimates of stunting, underweight and obesity in the El Alto adolescent sample produced by three different growth references: the widely used World Health Organization (WHO) reference [16–18], a Bolivian reference based on the Metabolic Syndrome in Adolescents (MESA) Study [19], and a recently published growth reference based on data collected from communities at high altitude in Peru’s Puno region [20].

One concern with rural to peri-urban migration, and more generally with *in situ* increases in the consumption of poor-quality processed food, is the heightened risk for the double burden of malnutrition. Monitoring the prevalence of poor growth outcomes is one of the tools used to combat this problem. However, interpretations of growth and adiposity patterns in any population depend upon the choice of a growth reference. The use of an inappropriate growth reference for a given population can lead to under-recognition of unhealthy growth and/or misdirection of resources toward addressing a perceived problem that may be more methodological than biological [21, 22].

The WHO growth reference for school-aged children [16–18] is widely used in clinical evaluations of individuals and epidemiological studies of populations. Questions have arisen over its global applicability, in part because the WHO growth reference is based on samples from only six countries, all below 1500 m altitude [22, 23]. Of particular importance, a foundational assumption of the development of the WHO growth reference is that under optimal conditions, all children share the same healthy growth trajectory. Some empirical evidence challenges this assumption including demonstrably different growth trajectories in healthy populations [22] and variation in body size and shape that may be evolved adaptations to climate and other features of the physical environment [21, 24–27].

However, as these and other authors have cautioned, it cannot be assumed that most variability in human growth is a consequence of adaptive mechanisms and should therefore be seen as ‘normal’ or, at a minimum, unavoidable. Such assumptions too readily ignore the unassailable reality of socially constructed barriers to the resources necessary for healthy growth [28–31] and also mischaracterize evolutionary processes.

Rather than necessarily being predetermined, evolved biological responses to both social and ecological challenges are often characterized by contingent trade-offs in the pace and magnitude of growth and reproduction. When challenges are few and resources are abundant, an individual can potentially grow to a larger size, better fend off disease, and have a relatively greater number of healthy offspring. But when resources are inadequate, individuals and their children are, metaphorically, making the best of a bad situation, trading off growth, reproduction, and health against each other. Faced with scarcity, the evolutionarily adaptive response may well not result in the healthiest state for the individual or their children [32–35].

There are several models regarding the evolved and proximate mechanisms that underlie these processes and outcomes [9, 36–42]. Although there are differences in the details, these and other evolutionary models emphasize that growth *in utero* and during childhood are major determinants of adult health. Even though ideal health is not possible [43], contemporary human well-being is less constrained by evolutionary history than by our willingness to implement a more equitable distribution of resources. Identifying growth patterns and morphological variation that arise from socioeconomic inequalities rather than from population-specific evolutionary adaptations to ecological challenges is likely to inform and improve efforts to reduce health disparities.

With this goal in mind, we evaluated the assessments of El Alto adolescents’ health, and the implications for suitable interventions (if any) that result from using these three different growth references. We expected the prevalence of stunting, underweight and obesity in the El Alto sample to differ depending upon the choice of a growth reference. Specifically, we expected the proportions of the El Alto sample classified as stunted and/or underweight to be higher if using the WHO growth reference (because it is based on well nourished, low-altitude populations), lower if using the MESA growth reference (because it is based on a representative sample of Bolivian children at all altitudes and economic conditions), and lowest if using the Puno growth reference (because it is based on samples from only high altitude communities).

As noted above, there is a concern that changes in dietary practices, particularly increasing consumption of processed foods, may increase overweight/obesity in urbanized populations. We therefore evaluated the prevalence of overweight/obesity in the El Alto sample. In light of the relatively scarce resources in the altiplano, we expected that the proportion of the sample classified as overweight/obese will be lowest if using the WHO reference, higher if using the MESA reference, and highest if using the Puno reference.

## 2. METHODS

### 2.1 Data collection

Data were collected during August and September 2003 from adolescents (11.0–14.9 years old, 60 females, 41 males) enrolled at a school principally serving low-income neighborhoods in El Alto. All protocols were approved by the Institutional Review Board at Northwestern University (IL, USA) and the Hospital San Gabriel (La Paz, Bolivia). For all study participants, parents gave informed consent for their children’s participation and adolescents gave assent.

A licensed Bolivian physician conducted a private medical examination that included body temperature, diastolic and systolic blood pressure, pulse, heart rate and recent medical history. Any child who was ill received appropriate medical treatment and discontinued participation in the study.

A bilingual (Spanish/Aymara) Bolivian researcher (E.C.) conducted a private interview with each study participant regarding current age, birthplace, residence duration in El Alto, extracurricular activities, parents’ occupations and languages, and recalled food intake over the previous 24 h.

The principal investigator (V.J.V.) measured all anthropometrics according to standard protocols [44]. Height was measured with a stadiometer, weight was measured with an SECA digital scale, and body mass index (BMI; equals (weight in kilograms)/(height in meters)^2^) was calculated from these measurements. Upper middle arm circumference (MAC) was measured with a non-stretch anthropometric tape. Harpenden skinfold calipers measured biceps, triceps, subscapular and suprailiac skinfolds. Each skinfold was measured three times and the mean of these was used in analyses.

### 2.2 Selection criteria and attributes of comparative samples

From the available published literature, we selected three previous community-based studies of children living at high altitude for comparison to the El Alto peri-urban sample. The selected studies had included adolescents of the same ages as the Alteño sample and had reported age- and sex-specific anthropometric descriptive statistics. To minimize any effects of a possible secular trend in growth in Andean populations, we selected those studies most recently antecedent to our study in El Alto in 2003. Three studies met these criteria: a study of urban children conducted in 1983 in La Paz at an altitude of 3750 m [12] and two studies of rural children, one conducted in 1977 in Ancoraimes, Bolivia, at an altitude of 3800–4000 m [11, 45, 46], and another conducted in 1998 in Marquiri, Peru, at an altitude of 4100 m [14, 47] (see ‘Supplementary Data: Fig. S1: Map of Study Communities’).

The La Paz sample, selected from two public schools in a lower-middle class neighborhood, comprised 446 children aged 10.6–19.8 years of which 13% had two Aymara surnames, 58% had two Spanish surnames, and 29% had one Aymara and one Spanish surname ([12]; Greksa 2021, personal communication). Their fathers were manual laborers (58%), held white-collar jobs (16%), or had various occupations (11%); 3% were unemployed, and 12% were deceased or retired. Most of the mothers did not work outside the home (72%); 11% were manual laborers, 9% were vendors, 5% had various occupations and 3% were deceased or unemployed. Only 30% of these parents, but 75% of the children, were born in La Paz, the rest having migrated from rural high-altitude villages. Of the sample of 446 La Paz students, there were 130 females aged 11.0–14.9 years and 136 males aged 12.0–14.9 years in the subsamples that we compared to the Alteño sample.

Ancoraimes is ∼3 km from Lake Titicaca, which makes the area suitable for growing potatoes, quinoa, beans and barley [11, 45]. This study sample included 510 Aymara children ages 6–20.9 years, all born at high altitude to parents who were native to the northern altiplano. Aymara was the presumed first language for all parents. As adults, only 20% of the mothers and half of the fathers also spoke Spanish. About 80% of the fathers earned most or all of their income from farming. Of the total sample, there were 55 females aged 11.0–14.9 years and 131 males aged 12.0–14.9 years.

Marquiri, Peru, is a rural village at ∼4100 m near the city of Yauri in the southern Peruvian Andes. At the time of the study, income was principally from part-time labor for largely unskilled tasks at the copper mining operation in nearby Tintaya; food and housing subsidies were non-existent, and there were no medical facilities in Marquiri [47]. This study sample comprised 442 predominantly Quechua or Quechua-Mestizo children (82% born at high altitude) aged 4–18 years, of whom there were 79 females aged 11–14.9 years and 53 males aged 12–14.9 years. Although children in Tintaya were also measured, we chose not to include this sample in the present analyses because only 60% of the measured children had been born at high altitude. (Supplementary Data: Section S3 provides additional context for Marquiri, a Peruvian Quechua community, and describes the ethnohistorical connections between Quechua and Aymara speakers.)

### 2.3 Selected reference samples

We compared the El Alto sample to three growth references: (i) the WHO growth reference for school-aged children [16–18], (ii) the MESA growth reference, based on a nationally representative sample of Bolivian children [19] and (iii) a recently published growth reference for high-altitude populations that is based on a sample from the Puno region of Peru [20].

The WHO growth reference for school-aged children is based on samples of infants and children from six countries ‘selected to be free of constraints to growth and hence growing optimally’ [17, 48].

The MESA study, undertaken to assess the risk for metabolic syndrome among Bolivian adolescents, collected data from 2005 to 2007 from a representative sample of 3445 adolescents recruited from 32 schools across the country [19]. The sample represents the altitudinal variation within Bolivia from the Andean highlands to the coastal lowlands. Approximately 34.8% of the sample were rural residents and 65.2% came from urban areas. The MESA authors explicitly noted that this sample is not a reference in the same sense as is the WHO reference (which is theoretically based on data from children who are growing optimally). Nonetheless, the data reflect the conditions country-wide in Bolivia at the time of collection.

The high-altitude growth reference proposed by Cossio-Bolaños *et al.* [20] is based on data collected during 2016 from the main school in each of 12 districts lying between 3821 and 4349 meters in Peru’s Puno region (not to be confused with the city of Puno). The Puno region includes most of the southern border region, with various agricultural, mining and tourist towns; 42% of households had access to potable water and 74% to electricity. Dietary diversity is low due to the very low temperatures that make agriculture difficult to sustain in many of the districts. Unlike the MESA sample, the Puno sample is not a representative sample *per se*; because the students are from ‘the lower middle class or emerging lower middle class’, it is likely they have not yet achieved the growth profiles that would be possible under more favorable socioeconomic conditions. This high-altitude reference sample comprised 1536 students, ages 5.0–17.9 years, of which 240 were females aged 11.0–14.9 years and 168 were males aged 12.0–14.9 years.

### 2.4 Analytical methods

Unless otherwise indicated, statistical evaluations of differences between samples were assessed with a Student’s *t*-test (alpha = 0.05). Dietary recalls were coded using NVivo 12 (QS International) and quantified to assess the most common foods and ingredients consumed. Statistical analyses were conducted using STATA 15 and SPSS *v*28. Sex- and age-specific (1-year age bins) descriptive statistics were calculated for anthropometric measurements.

#### 2.4.1 Comparing Alteños’ growth to rural and urban communities and to growth references

To compare children’s growth in El Alto relative to other high-altitude communities (specifically, Ancoraimes, Marquiri and La Paz), for each anthropometric we calculated the community-sex-age-specific z-score for each Alteño study participant based on the published statistics for each of these three communities: z = (i – x̄)/SD where i = El Alto child’s measurement, and x̄ and SD refer to a community’s sex-age-specific mean and standard deviation (SD), respectively, for that measurement. Community-specific, sex-specific mean z-scores for the El Alto sample were determined for each anthropometric.

We used the same procedure to calculate each El Alto child’s z-score with respect to the Puno reference and with respect to the Bolivian MESA reference. Because the MESA study published percentiles (rather than age-sex-specific means and SDs) for MAC, for each sex and 1-year age bin we approximated this anthropometric’s distribution by a Gaussian and estimated its mean and SD from a linear regression (straight-line fit) on a Q–Q plot. Z-scores were then calculated as described above. We used the online calculator developed by the Canadian Pediatric Endocrine Group (https://cpeg-gcep.shinyapps.io/who2007_cpeg/; last accessed 1 Sept 2022) to calculate El Alto z-scores with respect to the WHO growth reference.

#### 2.4.2 Evaluating hypothesized associations between Alteños’ growth and socioeconomic indicators

To standardize anthropometrics for growth-related differences across age and sex bins in the El Alto sample, analyses of potential socioeconomic correlates of adolescent growth used sex-age-specific z-scores calculated with respect to the distributions of anthropometric data collected in the study of rural Ancoraimes children [11, 45, 46] and sex-age-specific z-scores calculated with respect to the WHO growth reference. The use of a z-score (rather than the measured anthropometric) in these analyses allows us to combine the El Alto girls (or boys) regardless of age into a single sample. Any of the three community-based studies or three growth references could have served for calculating z-scores to be used in these analyses. Regardless of the selected standard, the association between a putative covariate and a sample of z-scores (for a given measurement of the El Alto children) would be very similar. We chose Ancoraimes for calculating z-scores because it is a large sample of rural Bolivian Aymara children who were born and lived at about the same altitude as the El Alto adolescents. We also calculated z-scores with respect to the WHO growth reference because of its widespread use in studies of children's growth, and because it includes references for BMI (which are not available in any of the community-based studies).

To evaluate the potential association of variation in adolescent growth with variation in economic circumstances, for each El Alto adolescent we constructed an index of relative household income (see ‘Supplementary Data: Section S4. Calculation of Household Income Index (HII)’). Because it would have been considered unacceptably invasive to inquire about monetary income, we relied on parental occupation (Table 1 and, for more details, Supplementary Table S4.3) to calculate HII. All assessments of occupations and calculations of HII were conducted blind to any data (e.g. anthropometrics) not directly used in the calculations of HII. The HII is treated as an ordinal variable in our statistical analyses.

**Table 1.** Parental occupations

	*n*	%
Mother’s occupation		
Office	3	3.0
Construction	1	1.0
Education	6	5.9
Retail/vendor	19	18.8
Food	23	22.8
Service job	3	3.0
Arts/crafts/musician	2	2.0
Housewife	44	43.6
Father’s occupation		
Office	10	10.6
Construction	22	23.4
Education	5	5.3
Transport	26	27.7
Retail/vendor	13	13.8
Food	5	5.3
Civil service	8	8.5
Service job	3	3.2
Arts/crafts/musician	2	3.1
No information/deceased	7	

Number and percent of either mothers or fathers engaged in each occupational sector. Income within each sector varies depending upon the specific job, and whether employment is part or full time and/or short or long term.

The primary unit of analysis is the household, rather than either parent alone, because an El Alto child typically experiences and grows in the context of a single household rather than in two contexts each comprising a separate flow of resources from each parent. Furthermore, in our study sample, 44% of the mothers did not have an occupation other than housewife and were not earning income. This arrangement may occur for either of two very different reasons: (i) because she does not have the time or skills that would allow her to earn income, and/or (ii) because her husband earns sufficient income for the household. Depending upon the community composition, the households of women not working outside the home may be over-represented among the poorest households (because the woman is not bringing in income) or among the richest (because she does not need to earn income). In either case, statistical analyses of an association of adolescent growth to the income stream of only one parent would not adequately represent economic variability in a sample or community. In addition, such analyses are vulnerable to Simpson’s Paradox (erroneous associations that emerge, reverse or disappear in the analyses of partitioned samples; see ‘Supplementary Data: Section S4.2’ for further discussion). For these reasons, the household is the primary unit of analysis in our assessment of an association between adolescent growth and household SES. Nonetheless, both ethnographic and quantitative evidence indicates much higher employment rates of El Alto mothers than existed in the comparative community samples (discussed in Section 4.3). Therefore, with the above caveats in mind, we have also examined the association between a mother’s income-earning occupation and her child’s growth (Supplementary Sections S4.1 and S4.2 details the analytical approach).

#### 2.4.3 Evaluating secular trends in growth

The four community-based studies compared in our analyses were collected at different times over a 26-year period. Therefore, anthropometric differences between these samples may reflect secular trends in growth in the Andean region as well as, or rather than, contemporaneous differences in living conditions in urban versus peri-urban versus rural settings. To estimate the effect of time (a proxy for direct measurement of potentially changing socioeconomic conditions over time), we used the following analytical strategy [49]. For each of the six samples (El Alto, Ancoraimes, Marquiri, La Paz, MESA and Puno), an overall mean z-score for height-for-age relative to the WHO reference (z-score^W^) was calculated as the average of the mean height-for-age z-score for girls and the mean height-for-age z-score for boys. Height was selected for this analysis because this anthropometric is a well-validated biomarker of socioeconomic conditions, so much so that it has come to be known as ‘the biological standard of living’ [50]. The annual change in mean z-scores^W^ between the two rural communities (Ancoraimes, measured in 1977, and Marquiri, measured in 1998) is an estimate of the effect of time on rural Andean children’s growth. The difference in z-score^W^ between La Paz in 1983 and the point on the rural trend line in 1983 is an estimate of the locality effects on Andean children’s growth in 1983. From these and other estimations, we evaluated the relative contributions of time (secular trend) and locality on El Alto adolescents’ growth.

## 3. RESULTS

### 3.1 Study sample description

The El Alto sample included 60 girls, ages 11.0–14.9 y and 41 boys, ages 12.0–14.9 y. Mean age (Table 2) was more than 4 months younger in the girls (two-sided *t*-test, *P* = 0.005). All of the children were born at and lived at high altitude (>3600 m). Ninety percent of the study sample were either born in El Alto or began residence there before 2 years of age; an additional five and six children had arrived by 5 and 9 years of age, respectively. Bolivian altiplano infants are typically breastfed on demand throughout the day, and sporadically through the night while co-sleeping, until two or more years of age [51, 52]. Commonly eaten soft foods (potatoes, broth) begin to complement nursing before the first birthday. Therefore, for both the infants born in El Alto and those arriving later, the most important nutritional resource in early life was very likely breastmilk. In all, 4 were born in rural communities and 47 in La Paz (because it is not uncommon for Alteños to access La Paz’s health services, giving birth in a La Paz facility does not necessarily equate with concurrent maternal residence in La Paz). Neither mean age nor sex distribution of the children differed between those born in El Alto versus elsewhere; mean age at arrival in El Alto did not differ by sex.

**Table 2.** Anthropometrics and biomarkers of El Alto adolescents

	Females	Males
	(*n* = 60)	(*n* = 41)
	x̄ ± SD	x̄ ± SD
Age^a^ (years)	13.1 ± 0.7	13.5 ± 0.7
Height (cm)	149.2 ± 5.8	151.6 ± 9.5
Weight (kg)	44.7 ± 7.4	42.7 ± 8.0
BMI (km/m^2^)	20.0 ± 2.4	18.5 ± 2.3
MAC (mid-arm circumference, cm)	23.1 ± 2.5	21.7 ± 2.5
Biceps skinfold (mm)	6.7 ± 2.6	4.6 ± 2.1
Triceps skinfold (mm)	15.1 ± 3.9	9.7 ± 3.7
Subscapular skinfold (mm)	12.0 ± 5.0	7.5 ± 3.8
Suprailiac skinfold (mm)	11.9 ± 5.5	7.3 ± 5.2
Sum of skinfolds (mm)	45.8 ± 15.4	29.1 ± 14.0
Body temperature (degrees Celsius)	36.9 ± 0.3	36.9 ± 0.3
Systolic blood pressure (mmHg)	106.4 ± 7.4	107.1 ± 7.2
Diastolic blood pressure (mmHg)	70.3 ± 7	70.9 ± 4.5
Pulse	78.1 ± 8.2	74.9 ± 11.6

a Age significantly differed between females and males; two-sided *t*-test, *P* = 0.005.

Participants’ diets (*n* = 100 for dietary recalls) mostly consisted of bread, soups and mixed dishes with potatoes, rice and/or noodles, vegetables and beef (see ‘Supplementary Data: Summary of Dietary Recall Data (Supplementary Table S5.1)’). Other commonly reported foods included salad, chuño (freeze-dried potatoes), chicken, tea and juice. Animal protein (beef, chicken) is typically only a few mouth-size pieces in a dish otherwise dominated by potatoes and starches. On the day of the interview, only one student had not eaten breakfast and three had not eaten lunch; eight had not eaten dinner the previous night. No student had skipped more than one meal. Thirty-one percent of the students reported not consuming any snacks; only 9% reported drinking soda in the previous 24 h.

Aymara is the dominant indigenous language in El Alto, La Paz, and the surrounding altiplano; Quechua speakers are relatively few in this region. Aymara or Quechua was the primary spoken language for 54% of the fathers, 63% of the mothers and 45% of the households (i.e. both parents or an unpartnered mother). Spanish was the primary spoken language for 41% of the fathers, 29% of the mothers and 25% of the households. Both Aymara (or Quechua) and Spanish were commonly spoken by 5% of the fathers, 8% of the mothers and 6% of the households.

At least two-thirds of the fathers were blue-collar workers, principally in transport (e.g. taxi and truck drivers, mechanics) and construction (e.g. bricklayers, carpenters). At most, another 16% held ‘grey’ collar jobs (e.g. highly skilled construction work, police). Only 15% held white-collar jobs (e.g. in offices, health and education; Table 1). As well as their considerable responsibilities in the home, more than half of the mothers earned income, principally by selling foods and goods (typically at stalls or makeshift set-ups in open markets). At most, 10% held white/grey-collar jobs; most of these women were educators.

Amongst mothers, the primary spoken language was significantly associated with holding a white/grey-collar job (Chi^2^ = 12.0, two-sided *P* = 0.017); 24% of those women who primarily spoke Spanish held white/grey-collar jobs. Of those mothers who primarily spoke Aymara or Quechua, only 4.6% held a white/grey-collar job; 53% earned income from blue-collar work.

In contrast, amongst fathers, there was no significant association between the primary language spoken and holding a white/grey versus blue-collar job. This finding is consistent with the greater Spanish fluency in rural and migrant Aymara and Quechua men than in women, likely a consequence of compulsory universal military service for Bolivian men.

Sex-specific mean anthropometrics and biomarkers are presented in Table 2. During an individual medical examination, none of the study participants had a temperature above 37.4 Celsius or a pulse above 100 beats per minute. All had systolic and diastolic blood pressures below the 95th percentile according to the international blood pressure references for adolescents [53].

### 3.2 Comparisons of El Alto sample to three selected high-altitude community samples

Table 3 lists the mean sex-age-community-specific z-scores for El Alto adolescents’ height, weight, MAC and triceps skinfolds. Sex-age-community-specific descriptive statistics for each anthropometric are given in ‘Supplementary Data: Tables S6.1–S6.5’.

**Table 3.** Mean sex-age-community-specific z-scores for the El Alto sample relative to three high-altitude community-based samples

	Ancoraimes 1977^a^	La Paz 1983^a^	Marquiri 1998^a^
Females			
z-Height	2.1	0.8	0.9
z-Weight	1.9	1.2	0.8
z-MAC	2.0	1.4	
z-Triceps			1.2
Males			
z-Height	2.0	0.9	1.2
z-Weight	1.7	1.3	1.0
z-MAC	1.9	1.3	—
z-Triceps			1.2

a Year of data collection.

The community-specific mean z-scores for the El Alto sample were at least 0.8 SD greater than the comparison sample for all anthropometrics in all three community studies (Table 3). For each of the community-specific comparisons, girls and boys had similar mean z-scores for a given anthropometric. These results suggest that the relative gains in absolute body dimensions in the El Alto sample have not been accompanied by much, if any, change in sexual dimorphism.

### 3.3 Associations of socioeconomic variables and adolescent growth

As described in Section 2, anthropometrics for the El Alto adolescents were standardized as age-sex-specific z-scores with respect to three high-altitude communities and three growth references. To evaluate hypothesized positive associations of growth and covariates, we used the anthropometric z-scores calculated with respect to the Ancoraimes sample (z-score^A^) and the WHO growth reference (z-score^W^).

The HII is an ordinal variable ranging from 0 to 7. Maternal spoken language and paternal spoken language are each binary categorical variables: lesser Spanish fluency (i.e. primarily speaks Aymara/Quechua) *v.* greater Spanish fluency (i.e. speaks Spanish equally or preferentially to Aymara/Quechua). As discussed earlier, we predicted a positive association between the anthropometrics of El Alto adolescents and indicators of household socioeconomic status (i.e. higher HII and greater Spanish fluency indicate higher socioeconomic status). Table 4 presents the significant associations of girls’ anthropometrics with HII (Spearman’s correlation analysis), or with parental language (Student’s *t*-test). For either z-score standardization, none of the adolescent boys’ anthropometrics were significantly associated with either HII or parents’ spoken language. Analyses excluding the seven children without language data for their father did not qualitatively alter the above findings. Birthplace was not significantly associated with any anthropometric variables in either sex.

**Table 4.** Significant associations of adolescent girls' anthropometrics and socioeconomic indicators

	Household income index		Maternal spoken language		Paternal spoken language	
	Spearman rho	p^c^	t	p^c^	t	p^c^
z-score^A^ for Alteño girls' **height**^a^	0.322	0.007	2.94	0.002	2.37	0.01
z-score^W^ for Alteño girls' **height**^b^	0.312	0.009	2.48	0.008	2.27	0.014
z-score^A^ for Alteño girls' **weight**^a^			2.68	0.005	1.88	0.033
z-score^W^ for Alteño girls' **weight**^b^			2.76	0.004	2.29	0.013
z-score^A^ for Alteño girls' **MAC**^a^			2.52	0.007	1.85	0.035
z-score^W^ for Alteño girls' **BMI**^b^			1.89	0.032		

a z-score^A^: anthropometric standardized to Ancoraimes sample.

b z-score^W^: anthropometric standardized to WHO growth reference.

c tests were one-sided; blank grey cells indicate that the hypothesized associations were not significant at alpha = 0.05.

We also evaluated the hypothesized association between adolescent growth and MII. Results are presented in Table 5 (analytical details are discussed in Supplementary Data: Section S4.2). Because the MII is an ordinal variable, we used a non-parametric test (the Jonckheere–Terpstra test) and controlled for paternal income by conducting separate tests for low, middle and high PIIs. Girls’ standardized anthropometrics (z-score^A^ and z-score^W^ for height and weight) were positively associated with MII for those households with low PII (all but one woman earned income) and for those households with high PII (9 of 15 earned income); z-score for MAC was also positively associated with MII in high PII households. In contrast, girls’ anthropometrics were not positively associated with MII in the middle PII households (19 of 33 earned income); it is likely that this outcome reflects Simpson’s Paradox (see Supplementary Data: Sections 4.1 and 4.2). Consistent with the analyses in Table 5, in low and high PII households, mean anthropometrics were higher in adolescents whose mothers earned income versus those who did not. However, the results for PII = middle are not consistent with the prediction that maternal income and adolescent growth are positively associated; these unexpected findings are presented and discussed in Supplementary Data: Section S4.2.

**Table 5.** Significant associations of adolescent girls' anthropometrics and Maternal Income Index (MII; stratified into Low, Medium, and High PII)

	Paternal Income Index = Low (0-2), n = 10			Paternal Income Index = Middle (3-4), n = 33			Paternal Income Index = High (5-6), n = 15		
	T_JT_^c^	z^d^	p^e^	T_JT_^c^	z^d^	p^e^	T_JT_^c^	z^d^	p^e^
z-score^A^ for Alteño girls' **height**^a^	29.0	2.317	0.01				68.0	2.581	0.005
z-score^W^ for Alteño girls' **height**^b^	29.0	2.317	0.01				64.0	2.17	0.015
z-score^A^ for Alteño girls' **weight**^a^	25.0	1.544	0.06				61.5	1.912	0.028
z-score^W^ for Alteño girls' **weight**^b^	24.5	1.453	0.07				56.0	1.34	0.09
z-score^A^ for Alteño girls' **MAC**^a^							57.5	1.5	0.067
z-score^W^ for Alteño girls' **BMI**^b^									

a z-score^A^: anthropometric standardized to Ancoraimes sample.

b z-score^W^: anthropometric standardized to WHO growth reference.

c Jonckheere-Terpstra test statistic.

d Standardized test statistic.

e Tests were one-sided; blank grey cells indicate that the hypothesized associations were not significant at alpha = 0.10.

Thirteen children reported missing one meal in the previous 24 h before being interviewed. These children did not differ from the other children in sex ratio, HII, or in the languages preferentially spoken by either parent. On average, these 13 children had significantly higher z-scores for weight (z-score^W^, *t* = 2.112, *P* = 0.037, two-sided), arm conference (z-score^A^, *t* = 2.357, *P* = 0.020, two-sided) and BMI (z-score^W^, *t* = 2.667, *P* = 0.009, two-sided) than did the children who had not missed a meal. However, the z-score^W^ for BMI in these 13 children ranged widely, from −0.8 to 1.9. According to WHO criteria, none were classified as underweight, and 6 were classified as overweight; weight classification was not significantly associated with sex (Fisher exact test: *P* = 0.27).

### 3.4 Comparisons of El Alto sample to three growth references

Table 6 lists the mean sex-, age-, and reference-specific (WHO, MESA or Puno) z-scores for the El Alto adolescents’ anthropometrics. Relative to the WHO reference, Alteño girls and boys were, on average, equally shorter (−1.1 SD). Mean z-scores for weight were also negative in both sexes, but more so for boys. Thus the mean z-score for BMI was negative in Alteño boys but positive for Alteño girls. Relative to the national Bolivian sample (MESA), Alteño adolescents were, on average, shorter and leaner (i.e. had relatively less adipose reserves for their sex and age). Leanness was more pronounced among the boys than the girls. In contrast, relative to the Puno high-altitude reference, Alteño adolescents, on average, had greater adiposity (more so in girls than boys) and were taller.

**Table 6.** Sex-, age- and reference-specific mean z-scores for the El Alto sample

	El Alto mean z-score with respect to growth reference		
	WHO	MESA 2005-2007	Puno 2016
Females			
z-Height	−1.1	−0.2	0.6
z-Weight	−0.4	−0.2	0.6
z-BMI	0.3	−0.1	0.3
z-MAC		−0.1	1.2
Males			
z-Height	−1.1	−0.3	0.7
z-Weight	−0.7	−0.5	0.4
z-BMI	−0.2	−0.5	0.1
z-MAC		−0.6	0.4

In sum, El Alto adolescents were, on average, relatively shorter and leaner than the WHO and MESA references but taller and had more adipose reserves than the Puno high-altitude reference sample. El Alto boys were consistently leaner, on average, than El Alto girls regardless of the growth reference used.

#### 3.4.1 Classification of individuals based on the WHO growth reference

Eight (13%) of the Alteño girls fell below −2 SD height-for-age (HFA), the WHO threshold for stunting, one of whom fell at −3 SD HFA (severe stunting is defined as <−3 SD HFA; Table 7). None of the girls had an HFA greater than +1 SD. Nine (22%) of the Alteño boys fell below −2 SD HFA, one of whom fell below −3 SD HFA (i.e. is severely stunted). Two boys had HFA greater than +1 SD. In total, 16% of the sample was classified as stunted and 1% was severely stunted.

**Table 7.** *N* (%) of El Alto sample categorized as stunted, underweight, overweight or obese using WHO, MESA and Puno growth references

	El Alto height-for-age	El Alto BMI-for-age		
	< −2SD	< −2SD	> +1SD < +2SD	> +2SD
	stunted	underweight	overweight	obese
Females				
WHO	8 (13%)	1 (2%)	10 (17%)	0
MESA	2 (3%)	0	5 (8%)	0
Puno	0	0	7 (12%)	2 (3%)
Males				
WHO	9 (22%)^a^	2 (5%)	5 (12.5%)	0
MESA	3 (7.5%)	0	1 (2.5%)	0
Puno	1 (2%)	0	3 (7.5%)	2 (5%)
Total sample				
WHO	17 (17%)^a^	3 (3%)	15 (15%)	0
MESA	5 (5%)	0	6 (6%)	0
Puno	1 (1%)	0	10 (10%)	4 (4%)

a One child is classified as severely stunted (<−3 SD).

One (2%) of the Alteño girls fell below −2 SD BMI-for-age (BFA), the WHO threshold for underweight. Ten (17%) of the girls had a BFA greater than +1 SD BFA (WHO threshold for overweight). Two (5%) of the boys fell below −2 SD BFA and five (12.5%) of the boys fell above +1 SD BFA. In total, 3% of the sample was classified as underweight, 15% was classified as overweight, and none of the Alteño adolescents fell above +2 SD BFA (WHO threshold for obese).

#### 3.4.2 Classification of individuals based on the MESA growth reference

We also classified each El Alto adolescent using the WHO threshold criteria applied to the z-scores calculated using the MESA growth reference (Table 7). Two (3%) of the girls and 2 (5%) of the boys had HFA z-scores between −2 SD (WHO threshold for stunting) and −3 SD (WHO threshold for severe stunting). One of the boys, but none of the girls, had a z-score below −3 SD HFA. In all, 4% of the sample was classified as stunted and 1% as severely stunted. Four (7%) of the girls and two (5%) of the boys had BFA z-scores above +1 SD (WHO threshold for overweight); 6% of the sample was classified as overweight. None of the Alteños had z-scores above +2 SD BFA (WHO threshold for obesity), and none had z-scores below −2 SD BFA (WHO threshold for underweight).

#### 3.4.3 Classification of individuals based on the Puno growth reference

Each El Alto adolescent was also classified using the WHO threshold criteria applied to the z-scores calculated using the Puno growth reference (Table 7). Only one of the Alteño boys and none of the Alteño girls had HFA z-scores below −2 SD (the WHO threshold for stunting). None of the Alteños had z-scores for BFA below −2 SD. Seven (12%) of the girls and three (7%) of the boys had BFA z-scores above +1 SD (the WHO threshold for overweight). Two (3%) of the girls and two (5%) of the boys had BFA z-scores above +2 SD (the WHO threshold for obesity). In total, 10% of the sample was classified as overweight, and 4% was classified as obese.

### 3.5 Estimation of secular trends in height in Andean communities

**Figure 1.**
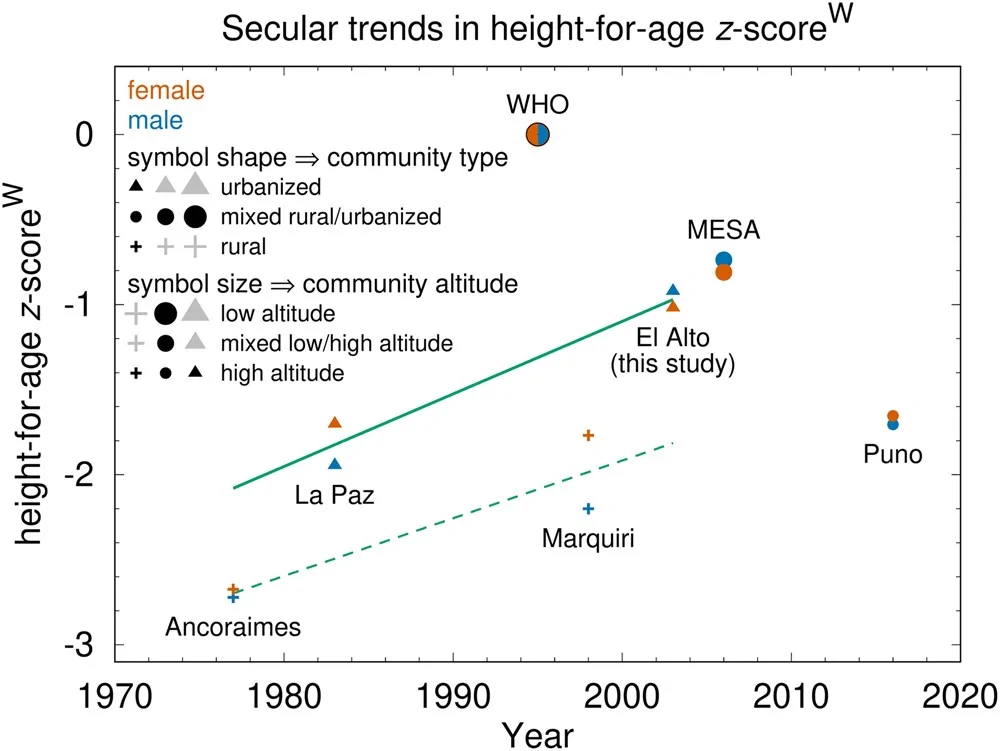
Secular trends in height-for-age (HFA) z-score^w^. z-score^w^ is calculated with respect to the WHO growth reference (see main text and Supplementary Data Section S7 for detailed explanation); mean HFA z-score^w^ is plotted for each of the seven samples examined in this article (symbol shape and size denote community type and altitude, respectively). Solid line depicts estimated urban HFA secular trend; dashed line indicates estimated rural HFA secular trend.

Peri-urban Alteños measured in 2003 were much taller than rural Ancoraimes adolescents measured in 1977 (Table 3). To estimate the portion of this difference that is potentially attributable to a regional secular trend in the altiplano regardless of specific locale versus the portion attributable to differences between contemporaneous rural and urbanized locales, we compared z-scores for height-for-age, explicitly taking into account the different years of data collection. Figure 1 depicts the effects on adolescent growth of time (i.e. regional secular trends in socioeconomic conditions) and of locale (rural vs urbanized). For each of the seven samples examined in this study, the sex-specific mean z-score for height-for-age relative to the WHO reference (z-score^W^ on the *y*-axis) is plotted against year of data collection (the *x*-axis). (Note: A step-by-step explanation of the following analyses is given in Supplementary Data: Section S7).

The increase over time in z-score^W^ between the two urbanized samples (La Paz and El Alto, which for the purpose of this analysis are both classified as urbanized in contrast to rural communities) reflects an urban secular trend in height; the slope (0.427 z-score^W^/decade) of the solid line is an estimate of the average urban secular trend in this region. The increase in z-score^W^ between the two rural samples, Ancoraimes and Marquiri, likewise reflects a rural secular trend in height; the slope (0.340 z-score^W^/decade) of the dashed line (Fig. 1) is an estimate of the average rural secular trend (Table 8). Note that each of these two slopes are only averages over two-plus decades, and should not be assumed to indicate a steady positive secular trend during these periods in either urbanized or rural communities. The slope of the mean secular trend equals 0.383 z-score^W^/decade.

**Table 8.** Determinations of secular trends and locality effects on adolescents’ height for age

	Secular trend	Height-for-age
	(z-score^W^/decade)	(z-score^W^)
Ancoraimes-to-Marquiri **rural trend line**: slope (z-score^W^/decade)	0.340	
La Paz-to-El Alto **urban trend line**: slope (z-score^W^/decade)	0.427	
**Mean secular trend**: slope (z-score^W^/decade)	0.383	
Urbanized Locality Effect (a): urban trend line value in 1977—Ancoraimes		0.617
Urbanized Locality Effect (b): La Paz—rural trend line value in 1983		0.670
Urbanized Locality Effect (c): urban trend line value in 1998—Marquiri		0.801
Urbanized Locality Effect (d): El Alto—Ancoraimes-to-Marquiri rural trend line in 2003		0.844
**Mean Urbanized Locality Effect (1977–2003)**		0.731
Total increase in mean height from Ancoraimes to El Alto		1.727
**Contribution of mean secular trend** (=0.383*2.6 decades)		0.996 (58% of total increase)
**Contribution of urbanized locality effect** (=1.727 − 0.996)		0.731 (42% of total increase)
Contribution of rural secular trend (=0.340*2.6 decades)		0.883 (51% of total increase)
Urbanized locality effect over and above rural secular trend (=1.727–0.883)		0.844 (49% of total increase)
Contribution of urban secular trend (=0.427*2.6 decades)		1.110 (64% of total increase)
Urbanized locality effect over and above urban secular trend (=1.727–1.110)		0.617 (36% of total increase)
MESA—La Paz-to-El Alto urban trend line		0.069

The effect of locality (urbanized vs rural) on adolescent growth was estimated as the average difference in z-score^W^ (0.731) between the rural and urban trend lines during the time span from the Ancoraimes sample (1977) to the El Alto sample (2003). The urban – rural difference in z-score^W^ at the times of the Ancoraimes, La Paz, Marquiri and El Alto samples is estimated to be 0.617, 0.670, 0.801 and 0.844, respectively (Table 8).

Thus, the increase in height (1.73 z-score^W^) of peri-urban Alteños in 2003 compared to rural Ancoraimes adolescents in 1977 can be attributed to a mean regional secular trend over 2.6 decades (about 58% of the total increase) and to the effect of peri-urban versus rural locality (about 42% of the total increase). If the secular trend had been as little as that estimated from rural communities’ data, the relative effects of these two factors on El Alto adolescents’ height would be about 51% due to the secular trend and 49% due to peri-urban locality. If the secular trend had been as high as that estimated from urban communities’ data, the relative effects of these two factors on El Alto adolescents’ height would be about 64% due to the secular trend and 36% due to peri-urban locality.

## 4. CONCLUSIONS AND IMPLICATIONS

The study of child growth and development in the Andean highlands has a long history, much of it directed to testing hypotheses of genetic and developmental adaptation to hypoxia, taking into account (to differing degrees) potential socioeconomic confounders. Some investigators have focused explicitly on the impacts on human well-being of differences between poorer and wealthier groups, nonetheless acknowledging that adaptive responses to high-altitude conditions likely make some contribution to growth trajectories and adult morphology [11, 12, 25, 45–47, 54–63].

Our approach was to examine the hypothesized effects of socioeconomic conditions on Andean adolescents’ growth at three levels of organization—between households within a peri-urban community undergoing rapid demographic and economic change, between different types of communities (rural, peri-urban, and urban), and over time in rural and in urbanized communities—while holding the effects of high-altitude constant by selecting only communities at about the same altitude.

### 4.1 Effects of household and community differences on adolescent growth

Anthropometric measures (z-scores for height, weight, BMI and MAC) of El Alto female adolescents were significantly positively associated with household socioeconomic status, operationalized in this study by the HII, which is determined from the parental occupations, and parental fluency in Spanish (which is significantly associated with women’s occupation level). We did not observe similar associations in the male adolescents, perhaps because of selection biases in the sample of boys (see discussion of study limitations below).

The study sample of peri-urban Alteños was, on average, taller and had greater energy reserves (adiposity) than their counterparts in previous studies in two rural communities at comparable altitudes. Although the sizable shifts in populations to urbanized settings are not necessarily an ideal solution to rural scarcities, our findings support the conclusions reached by O’Hare and Rivas [3], at least as regards the El Alto population, that rural out-migration to urbanized communities has been a viable poverty-alleviating strategy for some. Our findings also agree with those from Cockx [4], who demonstrated that in Tanzania the children of rural to urban migrants had greater height than children in the natal community, and with those from Andrissi *et al.* [64] who documented large differences in energy intake between urban and rural children in southern Peru.

### 4.2 Impacts of regional secular trends on adolescent growth

Peri-urban El Alto adolescents were substantially taller and heavier than urban La Paz adolescents measured 20 years earlier. Rural Marquiri adolescents were also substantially taller and heavier than rural Ancoraimes adolescents measured 21 years earlier. Overall, our analyses suggest that regional secular trends were a somewhat greater contributor than local peri-urban conditions *per se* to the improved growth in El Alto adolescents relative to rural Ancoraimes adolescents (Fig. 1 and Table 8). These secular trends in growth are consistent with the implementation of economic development programs in Bolivia, and in the Andes more generally [2, 3, 5, 15].

Although heterogeneous in magnitude, duration, locality and focus, in recent decades there have been significant socioeconomic improvements in various Andean communities. To differing degrees, these changes are associated with better growth and development of children from birth through adolescence [5, 47, 65–68]. As would also be expected, in the absence or loss of such economic improvements, secular trends in children’s growth will level or even reverse, as was observed in Nuñoa, Peru, during a lengthy period of political unrest and economic stagnation [14].

The Bolivian agrarian reform, which began in the 1960s, implemented resource and land grants with the goal of improving agribusinesses and economic opportunities for rural Bolivians [2]. In the 1990s, Bolivia undertook substantial development initiatives (mostly electricity, water and roads) in some of the poorest areas of the country. Our analyses found a modest difference between rural and urbanized localities in the estimates of the secular trends in adolescent height (slope for the rural trend line is 0.340 WHO SD/decade versus 0.427 WHO SD/decade for the urbanized trend line (Fig. 1 and Table 8)). But given the limitations of the data and regional variability in resources, it cannot be inferred from these analyses that rural development is generally less rapid than that of urbanized areas.

### 4.3 Women’s economic activities and adolescent growth

Bindon and Vitzthum [69] found that the nutritional status of rural altiplano Bolivian women improved with increasing reliance on westernized economic strategies (e.g. increasing education and employment). In the Peruvian altiplano, women’s control of home-based production of food (some of which she could sell) was positively associated with infant growth [70]. Studies in other populations living in marginal conditions have reported similar findings, and several (but not all) studies have reported positive associations between maternal employment and/or education and preadolescent children’s growth [67, 71–75]. More generally, children’s health is widely documented to be positively associated with improvements in the social and economic status of women [28, 30].

Such improvements may arise from a variety of economic activities that include, but are not limited to, participation in the formal economy. ‘Occupational multiplicity’ is a common strategy with a long history in Bolivia. Flexibility and ingenuity in combining income-generating activities with household and family obligations mitigate widespread economic precarity, especially if both parents contribute income to the household [76, 77]. In dual-income El Alto households, it was not necessarily the case that a wife supplemented a husband’s larger income. Of the two-parent households in our sample, 29% of the mothers had an income index equal to or greater than their husbands (see Supplementary Data: Sections S4.1 and S4.2). Regardless of the parents’ relative incomes, in an unstable job market it’s less likely that two working parents will simultaneously be unemployed than that one parent will be unemployed (i.e. any income generation by the lower-earning spouse is still better than none).

In Ancoraimes, paternal occupation was a significant covariate of children’s growth [11], but few women in this rural community worked outside the home (Stinson, 2021, personal communication). In Marquiri, most income at the time of the study came from part-time labor at a nearby copper mine [47]; although gender-specific occupation rates were not reported, such work in this region is rarely done by women.

In contrast to rural communities, urban and peri-urban localities typically provide a greater number and more diverse wage-labor opportunities for both men and women. About 56% of the mothers of the Alteño adolescents worked outside the home, including a few (10%) in white-collar jobs. The difference in economic structure in El Alto compared to rural natal communities is evident, and women as well as men take advantage of these opportunities. In the El Alto sample, we found that HII was positively associated with Alteño female adolescent height and adiposity.

Spanish language skills broaden the economic options in nearby La Paz as well as in El Alto. More mothers in the El Alto sample spoke Spanish than those in the Ancoraimes sample [45], and Spanish fluency in the Alteño mothers was associated with higher-paying employment. Maternal and paternal Spanish fluency were each positively associated with Alteño adolescent female height and adiposity.

In addition, regardless of the specific income level, year-round employment in El Alto—particularly of both parents—may lessen or even eliminate the seasonal variation in food availability common to Andean agropastoral communities. For example, regardless of household economic level, rural altiplano Peruvians had lower caloric intake and dietary quality during the preharvest versus the postharvest season [78], and in a predominantly agropastoral rural region about 100 km south of La Paz, early pregnancy loss was more than three times greater during the harshest seasons [79]. Peri-urban and urban households are generally better buffered from such seasonal food shortages, even if the income source changes during the course of a year.

Increasing maternal employment may have also contributed to the positive secular trend observed during the 20 years between the collections of the La Paz and El Alto samples. Only about one-fourth of the mothers of children in the La Paz study worked outside the home [12]. The 2-fold higher employment rate in El Alto mothers compared to those in La Paz may also reflect a less saturated and more accessible market for selling goods, foods and labor in this rapidly growing peri-urban setting.

Although the impact of maternal employment on the growth of Andean children and adolescents has not received much attention, all indications are that rural out-migration will continue, even if it slows. The effects of mothers' education and employment on their children’s growth in urbanized settings will likely grow in importance and should be more widely assessed.

### 4.4 Assessment of stunting, underweight and overweight in El Alto adolescents

In a hypothetical sample of children growing under the same ‘optimal’ conditions as the children in the WHO reference sample, the hypothetical sample is expected to have about 14% overweight children, and about 2% each of stunted, underweight and obese children. Because these categories are defined by the SD of the chosen reference distribution, if the same z-score thresholds recommended by WHO to define these four categories were to be used with an alternative growth reference (e.g. MESA), the expected proportions of the MESA sample in the four categories would be the same. However, the observed prevalence in a sample of interest (in the present case, the El Alto sample) might differ depending upon the population composition of the reference sample.

Based on WHO-derived z-scores, 17% of the Alteños were stunted/severely stunted (a prevalence nearly nine times higher than in the WHO reference) and 3% were underweight (1.5 times the WHO proportion). Using the MESA and Puno references, 5% and 1% of Alteños were classified as stunted, respectively. None of the Alteños were classified as underweight using either the MESA or Puno reference. As expected, the rates of stunting and underweight were highest when using the WHO reference, intermediate when using the nationally representative Bolivian MESA reference, and the lowest when using the Puno reference.

The prevalence of overweight was highest when using the WHO reference (15%, about the same as the WHO sample), intermediate when using the Puno reference (10%), and lowest when using the MESA reference (6%). Only use of the Puno reference classified any Alteños as obese (4%), a finding that requires cautious interpretation because of the lower-income status of the Puno sample. In their comparative analyses [20], the authors of the Puno study noted that the weight, height, and arm circumference of the Puno sample were comparable to those in the same La Paz sample that we have evaluated in this study. Our finding that growth in the Alteños in 2003 is substantially improved over that observed in La Paz in 1983 suggests that neither the La Paz sample (which was selected from two public schools in a lower-middle class neighborhood) nor the Puno sample represents optimal growth of Andean children. Nonetheless, both studies present valuable and otherwise unavailable data on the status of children in these high-altitude localities at a specific time.

A recently published growth study of nearly 9000 subjects (ages 4–17 years) drawn from three regions in Peru includes a high altitude (4107 m) sample from Junin in central Peru, about 1400 and 1700 km north from Puno and El Alto, respectively [80, 81]. The human development index and the per capita family income for Junin are the lowest of the study’s three samples [81]. Anthropometrics for the Junin sample had not yet been published by the time we had completed the analyses that we have reported here. Nonetheless, briefly comparing the mean anthropometrics from Junin [81] and Puno [20] for 1-year age bins from 11.0 to 14.9 is informative. The Junin girls are taller and heavier at younger ages but similar at the older ages. The Junin boys are modestly taller and heavier than the Puno boys at all these ages. These observations are consistent with the inference that there is not as yet a high-altitude sample that represents the growth of Andean children under reasonably optimal conditions (i.e. socioeconomic conditions comparable to those of the children included in the WHO growth reference).

#### 4.4.1 Growth retardation in El Alto adolescents

The debates regarding the suitability of the WHO reference for the evaluation of children’s growth in populations not represented in the WHO sample [22, 23] might prompt the inference that an apparently high prevalence of stunting in Alteños is largely an artifact of the chosen reference. The much lower prevalences of stunting in Alteños when either the Bolivia-wide MESA sample or the high-altitude Puno sample are used as the reference appear to be consistent with this inference.

However, there is not as yet a reference sample comprising indigenous Andean children who were born and matured at high altitude under otherwise healthy conditions. An admirable strength of the MESA reference is that it is based on a nationwide representative sample of Bolivian children. However, the authors [19] noted that the MESA reference ‘does not claim to be a standard that describes a population that followed healthy recommendations for all parameters that could affect normal growth and body composition, or a population that have developed its full genetic potential’. About half of the population of the Department of Puno, Peru, is poor or extremely poor [82]. The authors [20] of the proposed high-altitude standard noted that as of 2016 the Puno region from which they collected their data is ‘emerging from moderate poverty’, conditions which likely are not amenable for healthy growth in all children in this population. Therefore, the comparable prevalence of stunting in the Alteños and the Puno reference sample cannot necessarily be interpreted as a sign that Andean adolescents are experiencing desirable (i.e. healthy) growth rates.

There is compelling evidence that prenatal growth in indigenous Andean populations reflects evolved adaptations to high altitude that protect against the altitude-associated fetal growth restriction that is experienced by populations not indigenous to high altitude [42]. Furthermore, a study of potential gene–environment interactions between Andean ancestry and higher altitude suggests that adaptation to the physical environment plays a significant role in Andean children’s growth trajectories [25].

However, the evidence of a positive secular trend in height in this and other studies of Andean communities [47, 65] reinforces other findings that the relative shortness of Andean natives is not entirely, and perhaps not even principally, attributable to adaptation to high-altitude conditions. Absent evidence that these improvements in growth have plateaued, the collective findings argue that these high-altitude populations have not yet achieved their full genetic potential. The relative contributions of evolved responses and socioeconomic disparities to Andean children’s growth remain to be definitively determined. In any case, the prevalence of stunting in the El Alto sample, regardless of the chosen growth reference, indicates that scarcity and poverty continue to be significant impediments to children’s growth in the Andes.

#### 4.4.2 Adiposity in El Alto adolescents

Despite decades of research, there is an ongoing lively debate on the causes of the global rise in excessive adiposity (overweight and obesity), arguably the only common ground being that the etiology is likely multifactorial [83–89]. These controversies are beyond the scope of this paper, other than to note that there is understandable concern that the changes in diets and activities that accompany the adoption of more urbanized lifeways may contribute to increased adiposity and risk for serious chronic disease.

One pressing challenge in addressing these issues is how best to measure adiposity and define overweight/obesity in children in different populations. The most widely used approach is to compare a child’s BMI to the WHO sex-age-specific growth reference for BMI, which was developed with the intention that it be a suitable tool for evaluating all children in all populations [16, 17, 48]. Nonetheless, there are several persuasive arguments against assuming a single such standard is applicable to the entire human species [22, 23, 90–96].

Among the concerns relevant to assessments of Andean children is that BMI is sensitive to body proportions. A person with short legs relative to height (stocky skeleton) will have a higher BMI than a person with relatively long legs (slender skeleton), even though they have identical percentages of adipose tissue [21, 27, 92, 97]. Thus adiposity may be overestimated in persons and populations with body proportions stockier than those of the six populations that compose the WHO reference. The skeletal morphology of a large majority of indigenous Andean persons is stocky, perhaps due to cold adaptation and/or chronic hypoxia [24, 98, 99]. Several authors have questioned the suitability of the WHO reference for evaluating adiposity in Andean and short-statured persons [20, 21, 100, 101], and in adolescents more generally [102]. Inferences regarding the prevalence of overweight/obesity should take these caveats into account when using the WHO growth reference for evaluating adiposity in high-altitude children, particularly adolescents.

The relative portions of the El Alto sample classified as overweight using these three references did not confirm our initial expectations. In light of the generally impoverished conditions in El Alto, we had predicted that the use of the WHO growth reference would produce the lowest prevalence of excess adiposity and use of the Puno reference would produce the highest estimates (see Section 1.2). In fact, the El Alto sample has about the same portion of overweight children as the WHO reference sample and lower prevalences than if classified using either the Puno or MESA reference samples.

Collectively, these findings suggest that, despite living for all or nearly all of their lives in a peri-urban setting, excess adiposity (as assessed by BMI) is not unduly common in these Alteño adolescents. The prevalence of overweight is no higher, and that of obesity is much lower, than in the WHO reference sample, which comprises children growing in ‘optimal conditions’ [48]. Especially given the high levels of stunting in this population, and in light of the evidence that BMI is a poor indicator of excess adiposity in persons with stockier builds, it is reasonable to infer that the WHO growth reference overestimates the true prevalence of excess adiposity in Andean children. Our findings are consistent with the persuasive evidence and arguments offered by Baya Botti and colleagues [100] that the WHO growth reference and thresholds for overweight/obesity overestimate the prevalence of excess adiposity, and underestimate the prevalence of thinness, in a nationally representative sample of Bolivian adolescents (the MESA sample).

### 4.5 Trade-offs in socioeconomic development

Socioeconomic changes during recent decades have improved the lives and health of many Andean peoples, but these developments can have unintended negative consequences [2, 3, 8].

Many rural highland communities still do not have sufficient potable water, sewage disposal, electricity, social services and quantities and diversity of foods throughout the year. Income-generating work is often scarce. Disparities between these conditions and development in urbanized areas have contributed to increasing rural out-migration. Furthermore, the out-migration of younger adults appears to be exacerbating the poverty in these rural communities [2, 3]. At the same time, even though there was an increase in the percentage of the El Alto population that has its basic needs met, the absolute number of Alteños in poverty rose because of *in situ* population growth and the influx of migrants [3].

Adolescents’ activities in El Alto are likely less arduous than in rural communities where agricultural mechanization is rare, there are few roads (most of which are unpaved and often impassable in the rainy season) and little access to transportation [103]. In addition to walking some distance daily to attend school, rural children and adolescents typically have many chores such as pasturing sheep across a wide terrain, helping in the fields and caring for younger siblings [51]. The effects of these differences on physical growth can be substantial. For example, based on detailed reports of their own activities, the adolescents in the El Alto study sample had lower (*P* < 0.001) physical activity levels and total daily energy expenditure (TDEE) when sitting in school than if they had been engaged in the chores typical of traditional agropastoral adolescents [104]. The reduced TDEE (∼34 000 kcal less per year in the Alteño female adolescents), if not offset by reduced energy intake and/or increased growth in fat-free mass (e.g. skeleton, muscle), would potentially result in about 4.4 kg adipose gain per year. In the Alteño male adolescents, the TDEE reduction is ∼37 000 kcal less per year, potentially causing an adipose gain of 4.8 kg per year. The increased height in this Alteño sample, relative to their rural counterparts (Table 3), is consistent with the expectation that at least some of the calories not directed to chores were expended on increases in fat-free mass.

### 4.6 Study advantages and limitations

This study’s household-level analyses captured the variability within El Alto in household socioeconomic level and in children’s growth and, more importantly, the association between these variables. Analyses at more aggregated levels (e.g. wealthier neighborhoods vs. poorer neighborhoods) are less likely to identify the specific factors that impact children’s growth. Such specificity is valuable in developing more effective intervention programs. The dichotomous employment variable commonly used in large-scale surveys (e.g. mother does/does not work outside home) cannot fully disambiguate households in which a mother is not working outside the home because of a high-income earning partner from those households that are dependent on a single low wage because the mother cannot find outside work. The inability to capture this distinction may underlie studies that concluded children’s growth is healthier in households with non-employed mothers. More accurate evaluations of the impact of household economic variables on children’s health likely require the use of graded measures (such as the HII used in our study) that better capture economic variability.

The study sample was not a randomly selected representative sample of El Alto. There were one-third fewer boys than girls in the study sample; however, this was an accurate reflection of the population of children in our target age range in the selected school. A comparison of the distributions for the anthropometric z-score and household incomes of the study participants suggests that the ‘missing’ Alteño boys are biased to shorter/leaner (and likely poorer) boys. These boys may be working to help support their families rather than attending school; adolescent girls, even in poor families, are likely to be seen as more vulnerable (especially in a peri-urban setting compared to rural life) and hence safer in school than elsewhere. Boys may also be more valuable in the labor force than girls (i.e. a poor family could gain more income from a boy than from a girl in the labor force). This relative absence of shorter/leaner boys in the Alteño sample may be contributing to the lack of an association between socioeconomic variables and boys’ anthropometrics (unlike the significant positive association we observed in the girls).

Unfortunately, the study in El Alto had to be cut short because of escalating political unrest at the time. We were therefore unable to conduct in-depth interviews with the children’s parents and thus lack detailed information on the parents’ personal histories and economic activities.

### 4.7 Implications for future studies and health programs

Economic factors within and between communities are major determinants of Andean children’s growth that should be considered when evaluating the impact of, and human adaptations to, high-altitude environments. At the same time, the evolution of morphological and physiological adaptations to environmental challenges, and the consequent individual and population variability in growth trajectories, argue against the premise that a single growth standard is well suited to evaluating health disparities across all human populations. An unsuitable growth reference can lead to misclassification of healthy growth and/or under-recognition of unhealthy growth, with consequent misdirection of resources.

Depending on the purpose, national growth references may have some advantages over the WHO reference, but even national references may obscure significant regional and ethnic differences in growth, especially in ecologically diverse and/or middle- and low-income countries. If available, reporting study findings using both WHO and national/regional growth references can multiply the utility of the collected data at little additional cost. Decisions as to which are the better growth reference(s) can then be the choice of other investigators and readers, dependent on their question of interest (e.g. How well do the classifications predict health outcomes?).

Despite improvement, the Alteño adolescents remain, on average, below WHO and MESA medians for height and weight, and below the MESA median for BMI; adolescent boys are also below the WHO median for BMI. Unlike some lowland populations, the prevalence of overweight/obesity is not elevated in the El Alto sample.

Suitable interventions are clearly needed to reduce the prevalence of stunting in El Alto and in Andean rural communities and to concurrently prevent the rises in overweight and obesity that have been observed in other low-income countries. This need is especially acute in challenging and impoverished environments such as the Andean altiplano. As has been rightly noted by many [6, 105, 106], the problems of scarcity and excessive, but nutrient poor, calories are often intertwined and can both be ameliorated by the same measures: improving accessibility and consistent availability of sufficient nutritious food for all children.

The specific routes to accomplishing this goal will vary by culture, region, economic systems and country. The evidence for evolved responses to high altitude does not obviate the need for addressing the severe problem of stunted growth in Andean children, much of which is associated with socioeconomic disparities between households and communities. At the same time, the currently available genetic and growth data underscore the need for taking the altitudes of natal and childhood communities into account in growth monitoring.

Population-specific evaluations and interventions are more likely to alleviate disparities in children’s growth than a one-program-for-all approach. The findings of the present study suggest that increasing viable occupational and educational opportunities for Andean women in both rural and urbanized communities, in addition to providing basic utilities, health and social services, are very likely to have a significant positive effect on children’s growth and health. Monitoring growth trends is a useful tool that is best partnered with community- and household-level studies to detect and understand the specific factors, such as maternal employment, that cause or alleviate health disparities in different regions. Action can then be taken accordingly. This work remains to be done in Bolivia and much of the world.

## Supplementary data

Supplementary data is available at *EMPH* online.

## Supplementary Material

eoac033_Supplementary_Data

## Data Availability

Data underlying this article are available in the Dryad Digital Repository, at https://dx.doi.org/10.5061/dryad.000000063.
