# Supplementary material for: Socioeconomic impacts on Andean adolescents’ growth: Variation between households, between communities and over time
Source: Evol Med Public Health. 2022 Aug 22;10(1):409–28. doi: 10.1093/emph/eoac033 (PMC9454678; doi:10.1093/emph/eoac033)
Supplement: eoac033_Supplementary_Data [file eoac033_supplementary_data.pdf]

SUPPLEMENTARY DATA

"Socioeconomic Impacts on Andean Adolescents' Growth: Variation Between Households, Between Communities, and Over Time"

Ms. Number: EMPH-2021-145; *Corresponding author:* VJ Vitzthum (vitzthum@indiana.edu)

S1. Study Setting: El Alto, Bolivia

Peri-urban communities are often described as municipalities with lower population density lying at the periphery of major cities, but this simple definition doesn't convey the many functions nor the variation within and between peri-urban localities [1,2]. Throughout its history and into the mid-twentieth century, El Alto had few residents [3]. Its spacious and flat terrain served better for the airport and a few other industries than did the contiguous steep canyon that is home to La Paz, the country's executive capital. But at an altitude of about 4150m, El Alto's harsher climate discouraged much human settlement. In 1950, Paceños (La Paz residents) outnumbered Alteños (El Alto residents) about 30 to 1 (321,063 versus 11,000). By 2012 (within only 60 years), Alteños exceeded Paceños, principally a consequence of indigenous Aymara rural to urban migration prompted by local and national economic and climatic factors that were and are paralleled globally [3]. In 2006, El Alto's population was approximately 830,000, with 74%-80% identifying as indigenous Aymara [4,5].

The complete disappearance in 2009 of the 18,000-year-old glacier on Chalcaltaya, only 30 kilometers from La Paz [6], is but one indicator of the environmental changes that continue to motivate rural out-migration. Climate change has exacerbated the already harsh conditions of the Andes (e.g., cold temperatures, aridity, water scarcity, poor soil conditions [7,8]) causing more droughts, increased erosion, longer dry seasons, fewer rain days but torrential downpours, and a mix of increasing temperatures paired with periods of frost and hail [9]. Thus, climate change has rendered rural economic strategies less tenable and driven individuals and families to migrate to urbanized areas in search of employment [9,10].

Legally incorporated as a city separate from La Paz in 1987, El Alto is functionally a critical transition zone between densely populated La Paz and the altiplano's sparsely populated rural regions, most of which are only occasionally crossed by roadways connecting cities and countries [3]. El Alto's nearly unfettered, but uneven, sprawl across the altiplano landscape allows for a less dense population than in La Paz but is accompanied by far fewer services. Although El Alto is economically diverse, with some better-off neighborhoods, generally those districts further from La Paz are poorer, having haphazardly come into existence with the arrival of new migrants.

In the 1990's Bolivia undertook infrastructure development initiatives for new water and sewage connections, and new electricity and telephone services, in the poorest neighborhoods of El Alto and La Paz [11]. Despite these improvements, the poverty rate in El Alto was 58.5 percent in 1999 [11]. A 2001 report noted that only 7% of Alteño households had all basic necessities met; 54% relied on outdoor water sources, and 37% did not have access to a toilet or latrine [3]. According to a report by the Institute for Advanced Development Studies, El Alto in the early 2000s was one of the poorest cities in Bolivia [12]. Bolivia, in general, is considered to be the poorest country in South America [10].

Today, La Paz and El Alto together constitute the second-largest metropolitan area in Bolivia. Occasionally referred to as "twin cities," El Alto and La Paz are far from identical. Although well over 100,000 Alteños commute daily to La Paz for work, much of the El Alto economy and infrastructure relies on small family-run businesses and services [13]. The wealth disparity between La Paz and El Alto is plainly evident. For example, circa 2000, the tax revenues per capita in La Paz were about five times greater than those in El Alto [3]. Consequently, in La Paz there are more social services including healthcare and education, and potentially more and/or better-paid work opportunities, than in El Alto.

However, urban settings can also be detrimental for migrants. Congestion, pollution, crowded housing, inadequate sanitation, and loss of traditional support networks pose new threats to migrant health and well-being. As individuals enter the non-agricultural workforce and children spend more time in school [14], increased sedentism and greater availability of poor quality energy-dense foods (a consequence of the globalization of food markets) may raise the risk for obesity and other non-infectious diseases [15]. Although urban Andean children grow taller and have better nutritional status compared to rural Andean children [16–18], some studies have reported a rise in the rates of overweight, obesity, and anemia among urban Andean children [19–21].

Thus, despite economic disparities between La Paz and El Alto, peri-urban districts may be a more viable destination for rural migrants than would an urban center. It is uncertain whether the documented disadvantages of urban environments also necessarily characterize peri-urban settings or if the effects of these problems are mitigated by, for example, lower population density and/or less expensive housing and food. Peri-urban settings may also be better positioned, literally, to allay food scarcity without promoting reliance on processed "obesogenic" foods. Produce from outlying agricultural sources may be more variable and more consistently available in peri-urban communities than in some rural migrants' natal villages, and also more affordable than in an urban center. Therefore, under such conditions, migrant children in peri-urban communities may grow better than their counterparts in urban and rural communities. Rural family members may also benefit from the goods and foods that rural to peri-urban migrants bring on visits to their natal communities.

In choosing between urban and peri-urban residence, considerations likely to be particularly important to Bolivian altiplano rural out-migrants, and arguably to rural out-migrants elsewhere, are housing costs, proximity to natal rural communities, which facilitate maintenance of core social and economic ties, and access to urban social services and economic opportunities. Because El Alto collectively serves these multiple goals better than does La Paz, El Alto may be more attractive to rural migrants than La Paz despite the capital city's relatively greater wealth and services.

S2. Map of Study Communities (Figure S1)

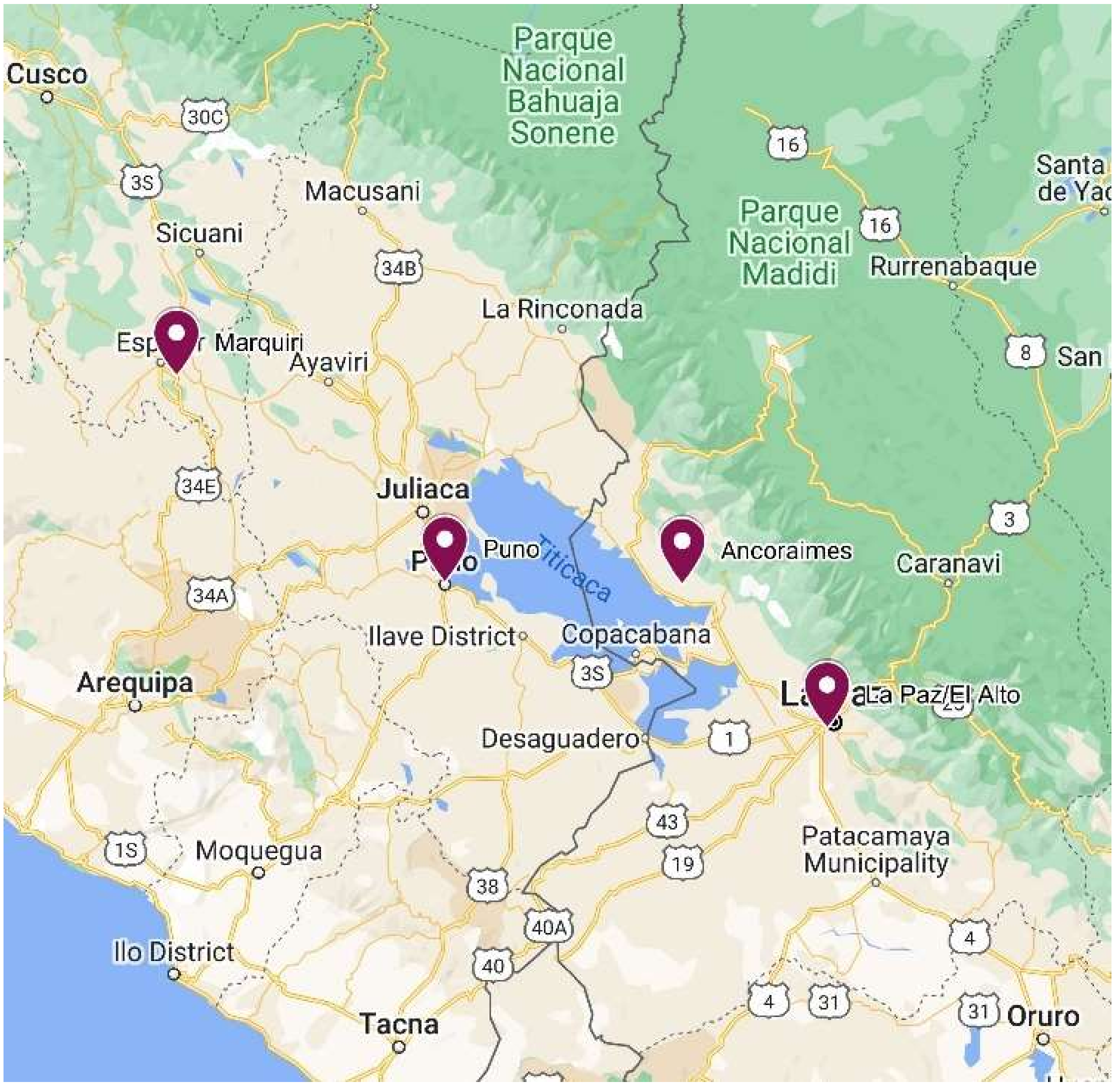

Map of La Paz & El Alto, Bolivia; Ancoraimes, Bolivia; Marquiri, Peru; and the Puno region of Peru.
Map data©2022 Google

S3. The Marquiri (Peru) Sample; Quechua-Aymara Ethnohistory

For our analyses, of the options available in the published literature, we selected the Marquiri sample collected in 1998 and reported in Pawson et al. 2001 [22] because (a) it included anthropometric data by age and sex for the same ages as the El Alto sample, (b) the data were collected in 1998, close to the time of data collection in El Alto in 2003, (c) 82% of the children in the Marquiri sample had been born at high altitude, and (d) comparative analyses by Pawson and Huicho [23] suggested Marquiri was neither the most disadvantaged nor the most advantaged rural high altitude Andean community at about the time the El Alto sample was collected.

Hoke and Leatherman [24] published a comprehensive assessment of secular trends in Nuñoa, Peru, (which is about 200 km SW by road from Marquiri) in which they cited Pawson and Huicho [23], who had collected data from Nuñoan children in 1999 and compared the sample to Nuñoan children in 1964 and Marquiri children in 1998 [22]. Pawson and Huicho [23] found that there was little difference in height-for-age (HFA) in Nuñoa children in 1964 (mean z-score = -2.33) and 1999 (mean z-score =-2.21). The Marquiri HFA mean z-score in 1998 was substantially higher (-1.83) than both the 1964 and 1999 Nuñoa samples. Pawson and Huicho [23] attributed these differences to the relatively greater political unrest and socioeconomic hardship in Nuñoa than in Marquiri. In Pawson et al. 2001 [22], the authors compared the 1998 Marquiri sample to a contemporaneous sample of children from Tintaya, a nearby mining-supported community. Reflecting the better conditions, Tintaya children were taller and heavier than Marquiri children.

The contemporaneous (c. 1998-99) predominantly Quechua samples from Marquiri, Tintaya, and Nuñoa exemplify the variation in growth across Andean rural locales at similar altitudes. Pawson and Huicho [23] argued that these differences are reasonably attributable to differences in living conditions that are a consequence of economic, social and political factors that were particularly exacerbated in Nuñoa.

The relationship between Quechua and Aymara speakers has been a focus of many genetic, linguistic, and archeological inquiries over several decades (see, for example, [25–29]). The picture is complex, and there are at least two primary models, neither fully supported, of dispersion of peoples from lowland to the Andean highlands [28].

In brief, according to Rothhammer [26] and papers cited therein, linguists agree that Aymara and Quechua are closely related languages. Although there are some genetic differences between different groups of speakers of these two languages, it has also been argued that some Quechua and Aymara groups display less difference between them than do some Aymara groups (e.g., Goedde et al. [30] reported that the genetic distance between the groups was not statistically significant). These patterns may reflect cultural imposition of the Quechua language on non-Quechua speakers as the Inca Empire spread, and perhaps also resistance to the Empire by Aymara speakers in the Titicaca Basin [27].

To the best of our knowledge, any differences between Quechua and Aymara speakers in genetic factors that may affect growth in these two indigenous high altitude populations appear to be much less influential than variation in socioeconomic and political conditions. De Meer et al. [8] reached the same conclusion in their analyses of Quechua and Aymara children in southern Peru. Likewise, in a review of variations in body size and shape among South American Indians, Stinson (1990) [31] suggested that patterning in body size and shape is greater than could be accounted for by genetic variation.

S4.1 Calculation of Household Income Index (HII)

Because it would have been considered invasive in the study setting to ask about monetary income, parents' occupations were used to construct the household income index (HII).

The HII is a relative ranking that operationalizes the overall household economic status based on the primary occupations of the student's parent(s); every student lived in a household with at least one parent. The HII is an ordinal variable ranging from 0 to 7; as explained below, HII values have both integer and fractional parts.

All occupation rankings and the construction and calculation of HII were conducted blind to any data (e.g., anthropometrics) other than parental occupations. Maternal and paternal occupations were ranked separately, without a household identifier or any reference to the other spouse's occupation. Occupations were sorted into categories corresponding to different economic sectors (Table S4.3); within each sector, occupations were ranked and scored to define an income index (II) ranging from 1 (lowest) to 6 (highest) based on relative income, social status, and precariousness. In addition to the relative ranking of jobs within a sector, ranks were assigned so as to be roughly comparable across sectors. An unemployed parent was assigned an income index of 0. These rankings were based on the ethnographic experiences (observations and conversations with collaborators and community members over many years in various altiplano communities including El Alto) of the lead author (VJV) and an economic anthropologist (LS), each of whom has worked extensively in the Andes since the 1980s.

For the class structure and economic conditions of El Alto, summing the father's and mother's income indices (i.e., defining HII = II_father + II_mother) is likely an over-estimate of household economic status. For example, an El Alto household with II_father = 3 (e.g., locksmith; see Table S4.3) and II_mother = 2 (e.g., maid) likely has significantly less social status, less income, and is less secure than a household with II_father = 5 (e.g., mining driller) and II_mother = 0 (unemployed). On the other hand, because a second income does improve a household's economic status, a household with II_father = 3 (locksmith) and II_mother = 2 (maid) has significantly more, and more stable, income than a household with II_father = 3 (locksmith) and II_mother = 0 (unemployed). Two incomes also mitigates against precarity.

A given second income is more valuable in a poorer household than in a better-off household. For example, the improvement in overall household economic status resulting from a mother transitioning from unemployed (II_mother = 0) to working as a maid (II_mother = 2) is larger for a household where the father is a locksmith (II_father = 3) than for a household where the father is a welder (II_father = 4) or a mining driller (II_father = 5). (In economics this concept is known as the "diminishing marginal utility of income".)

Reflecting this, we have defined the HII to be the income index of the higher-income-index spouse, plus an additional "bonus" to reflect the contribution of the lower-income-index spouse if s/he is also employed. (In this sample there were no households where both spouses were unemployed.) The bonus depends on the difference between the spouses' income indices: the bonus is +1.0 for a difference of 0, and decreases linearly with the difference to a bonus of 1/6 for a difference of 5 (the largest possible difference given that the lower-income-index spouse is employed). In other words, the effect on overall household income and stability of adding a second parent with the same income index as the first parent is roughly equal to that of moving up 1 point on the income-index scale. If instead the second parent has a lesser income index

than the first, then the effect on overall household income and stability will be less than that for the equal-income-index case; how much less depends on the degree of difference between the two parents' income indices (Table S4.1).

Table S4.1: Bonus for each degree of difference between spousal incomes.

absolute value of difference $\|II\_father - II\_mother\|$	bonus
0	1.00
1	0.83
2	0.67
3	0.50
4	0.33
5	0.17

This table shows the bonus for the case where both parents are employed. If only one parent is employed, there is no bonus.

For example, given spouse job scores of 3 and 1, HII = 3.67. This adjustment reflects the premise that the baseline HII (which equals the job score of the highest earner) is substantially increased by a second income that is equal to that of the highest earner (thus increasing the baseline HII by 1) and only minimally increased by a second income that is much lower than that of the highest earner.

In summary, we define the HII as HII = max(II_father, II_mother) + bonus

where

$$\text{bonus} = \begin{cases} 0 & \text{if } \min(\texttt{II\_father}, \texttt{II\_mother}) = 0 \\ 1 - \frac{1}{6}|\texttt{II\_father} - \texttt{II\_mother}| & \text{otherwise} \end{cases}$$

Table S4.2. The HII for each possible combination of mother's and father's income indices.

II_father → II_mother ↓	0	1	2	3	4	5	6
0	0.00	1.00	2.00	3.00	4.00	5.00	6.00
1	1.00	2.00	2.83	3.67	4.50	5.33	6.17
2	2.00	2.83	3.00	3.83	4.67	5.50	6.33
3	3.00	3.67	3.83	4.00	4.83	5.67	6.50
4	4.00	4.50	4.67	4.83	5.00	5.83	6.67
5	5.00	5.33	5.50	5.67	5.83	6.00	6.83
6	6.00	6.17	6.33	6.50	6.67	6.83	7.00

Table S4.3. Occupational Categories and Ranks with Examples of Occupations

INCOME INDEX RANK	Examples of occupations in each category
MIDDLE-HIGH [R=6]	business owner, engineer
MIDDLE-A [R=5]	bank cashier, butcher, mechanic
MIDDLE-B [R=4]	plumber, salaried driver, secretary, teacher
MIDDLE-C [R=3]	food vendor, locksmith, store clerk, taxi diver
LOW-A [R=2]	gardener, knitter, maid
LOW-B [R=1]	general hard labor, occasional vendor

S4.2 Analyses of Maternal Income Index (MII)

About twice as many El Alto mothers worked outside the home as did La Paz mothers; in Ancoraimes very few mothers worked outside the home, and in Marquiri there was little employment for men and likely much lower employment for women. It is logical to expect the addition of maternal income in El Alto households would positively impact children's growth.

However, it is challenging to statistically evaluate the effect of increasing maternal income because doing so requires controlling for variation in the paternal income index (PII) (i.e., holding PII constant). Because the income variables in this study are ordinal rather than continuous, standard regression techniques cannot be used.

Also, in the Alteño sample, the PII is usually, but not always, larger than the MII (larger: n=67; equal: n=16; smaller: n=11; no information on paternal and/or maternal occupation: n=7. Of the total sample, 44% of the mothers were not earning income, and in 29% of the dual-earner households, the mother had an equal or higher income index than the father. A mother may not earn income because (a) because she does not have the time or skills that would allow her to earn income, and/or (b) because her husband earns sufficient income for the household.

In the households with the poorest fathers (PII ≤2), 72% of the mothers were employed outside the home, whereas in the households with the higher-income fathers (PII ≥3), only 55% of the mothers were employed outside the home. In other words, the subsample of households where mothers are employed outside the home overrepresents poor households. This means that a univariate analysis of the effects of mother's income as a sole predictor of children's growth would tend to under-estimate or even reverse the actual effect due to Simpson's Paradox (erroneous associations that emerge, reverse, or disappear in the analyses of partitioned samples).

To address these issues, we used a non-parametric test (the Jonckheere-Terpstra test) and controlled for paternal income by conducting separate tests for low, medium and high PII. Alpha was set at 0.10 because of the smaller sample sizes in each PII bin; outcomes that do not meet this threshold are greyed in Table S4.4, which presents the findings. The results for PII=Low and PII=High are consistent with the prediction that MII makes a significant contribution to improving El Alto adolescents' growth.

In the case of PII=Middle, the results do not support the prediction because the z-statistic is negative (i.e., greater maternal income appears to *worsen* offspring growth) at p<0.10 for four variables. This outcome is sufficiently illogical, especially in light of the results for higher and lower PII, that it is arguably more parsimonious to suspect that this outcome is a case of Simpson's Paradox, perhaps due to the diversity of paternal occupations in the "PII=middle" subsample. There are also several unknown factors that may underlie this paradox including unreported (to us) economic activities in the informal sector and/or temporary or longer-term unemployment.

Table S4.4. Significant associations of adolescent girls' anthropometrics & Maternal Income Index (for Low, Middle, and High Paternal Income Index subgroups)

	Paternal Income Index Low (0-2), n=10			Paternal Income Index Middle (3-4), n=33			Paternal Income Index High (5-6), n=15		
	T_{JT}	z	p*	T_{JT}	z	p*	T_{JT}	z	p*
z-score[A] for Alteño girls' **height**	29.0	2.317	0.01	177.0	-0.894	0.19	68.0	2.581	0.005
z-score[W] for Alteño girls' **height**	29.0	2,317	0.01	161.5	-1.397	0.08	64.0	2.17	0.015
z-score[A] for Alteño girls' **weight**	25.0	1.544	0.06	143.5	-1.982	0.023	61.5	1.912	0.028
z-score[W] for Alteño girls' **weight**	24.5	1.453	0.07	152.0	-1.706	0.044	56.0	1.34	0.09
z-score[A] for Alteño girls' **MAC**	22.0	0.965	0.17	142.5	-2.015	0.022	57.5	1.5	0.067
z-score[W] for Alteño girls' **BMI**	19.0	0.386	0.35	175.0	-0.958	0.17	52.0	0.93	0.18

*[*tests were one-sided; grey cells indicate that the hypothesized associations were not significant at alpha=0.10]*

S5. Summary of Dietary Recall Data (Table S5.1)

Dietary recall counts and percent of sample for the top-ten most consumed food items per meal

Breakfast	n	%	Lunch	n	%	Dinner	n	%	Snacks	n	%
bread	93	93%	potatoes	78	78%	potatoes	55	55%	nothing	31	31%
tea	41	41%	soup	64	64%	rice	50	50%	juice	14	14%
milk	22	22%	rice	56	56%	beef	35	35%	orange	11	11%
chocolate	21	21%	vegetables	52	52%	soup	31	31%	soda	9	9%
coffee	15	15%	beef	50	50%	vegetables	27	27%	plantain	9	9%
cheese	9	9%	noodle(s)	39	39%	noodle	24	24%	bread	8	8%
egg	4	4%	meat	23	23%	meat	18	18%	apple	6	6%
			salad	22	22%	chuño	12	12%	potatoes	5	5%
			chuño*	18	18%	chicken	11	11%	cheese	5	5%
			chicken	12	12%	bread; salad	10	10%	yogurt	4	4%

*chuño = freeze-dried potatoes

S6. Anthropometric Descriptive Statistics (Tables S6.1-S6.5)

Sources for data in following tables: Ancoraimes [32], **La Paz** [16], **Marquiri** [22], **MESA** [33], **Puno** [34]

Table S6.1. Height (cm) by age and sex for high-altitude community & reference samples (with date of data collection)

	Ancoraimes 1977		La Paz 1983		Marquiri 1998		MESA 2005-2007		Puno 2016	
	n	x̄ ± SD	n	x̄ ± SD	n	x̄ ± SD	n	x̄ ± SD	n	x̄ ± SD
**Females**										
11-11.9	17	128.9 ± 4.2	16	136.7 ± 8.6	21	133.8 ± 7.6			79	136.7 ± 6.8
12-12.9	13	136.4 ± 6.3	41	142.6 ± 4.7	23	141.3 ± 5.8	170	149.1 ± 7.4	64	141.9 ± 6.5
13-13.9	14	138.9 ± 5.2	31	146.0 ± 6.6	24	146.5 ± 5.6	214	152.1 ± 7.9	54	147.6 ± 5.7
14-14.9	11	140.6 ± 4.9	42	150.2 ± 5.2	11	145.6± 4.0	265	153.3 ± 5.7	43	150.8 ± 5.6
**Males**										
12-12.9	36	133.4 ± 6.1	57	138.0 ± 5.6	15	137.6 ± 6.2	172	147.6 ± 8.7	63	140.0 ± 6.6
13-13.9	37	138.3 ± 6.7	45	145.4 ± 6.8	15	141.8 ± 7.5	221	153.7 ± 8.2	55	146.8 ± 6.5
14-14.9	58	144.6 ± 6.9	34	153.4 ± 8.4	23	152 ± 8.1	221	160.9 ± 8.0	50	153.9 ± 8.8

Table S6.2. Weight (kg) by age and sex for high-altitude community & reference samples (with date of data collection)

	Ancoraimes 1977		La Paz 1983		Marquiri 1998		MESA 2005-2007		Puno 2016	
	n	x̄ ± SD	n	x̄ ± SD	n	x̄ ± SD	n	x̄ ± SD	n	x̄ ± SD
**Females**										
11-11.9	17	29.8 ± 2.9	16	31.8 ± 5.6	21	30.6 ± 5.3			79	32.6 ± 6.2
12-12.9	13	33.5 ± 4.5	41	36.1 ± 4.7	23	37.2 ± 6.4	170	44.4 ± 9.4	64	35.7 ± 9.2
13-13.9	14	37.0 ± 6.7	31	40.4 ± 5.6	24	41.6 ± 6.8	214	47.6 ± 7.8	54	42.8 ± 8.3
14-14.9	11	41.2 ± 3.97	42	45.0 ± 6.9	11	43.4 ± 7.2	265	51.0 ± 8.4	43	45.1 ± 6.5
**Males**										
12-12.9	36	31.8 ± 3.9	57	32.1 ± 3.6	15	33.6 ± 4.7	172	43.8 ± 9.9	63	34.5 ± 7.7
13-13.9	37	34.8 ± 4.5	45	36.1 ± 4.8	15	36.0 ± 5.4	221	47.4 ± 10.0	55	39.8 ± 5.8
14-14.9	58	39.4 ± 5.6	34	42.8 ± 7.2	23	43.2 ± 6.3	221	52.6 ± 10.0	50	43.8 ± 8.1

Table S6.3. Body Mass Index [BMI] (kg/m2) by age and sex for reference samples (with date of data collection)

	MESA 2005-2007		Puno 2016	
	n	x̄ ± SD	n	x̄ ± SD
**Females**				
11-11.9			79	17.4 ± 2.6
12-12.9	170	19.8 ± 3.2	64	17.5 ± 3.6
13-13.9	214	20.6 ± 3.1	54	19.6 ± 3.1
14-14.9	265	21.7 ± 3.3	43	19.8 ± 2.4
**Males**				
12-12.9	172	19.9 ± 3.4	63	17.5 ± 3.0
13-13.9	221	19.9 ± 3.2	55	18.4 ± 2.2
14-14.9	221	20.3 ± 2.9	50	18.4 ± 2.1

Table S6.4. Mid-arm Circumference [MAC] (cm) by age and sex for high-altitude community & reference samples (with date of data collection)

	Ancoraimes 1977		La Paz 1983		MESA 2005-2007		Puno 2016	
	n	x̄ ± SD	n	x̄ ± SD	n	x̄ ± SD	n	x̄ ± SD
**Females**								
11-11.9	17	17.8 ± 1.4	16	18.9 ± 1.6			79	18.9 ± 1.7
12-12.9	13	18.8 ± 1.3	41	20.0 ± 1.6	170	22.8 ± 2.7	64	20.1 ± 1.9
13-13.9	14	19.8 ± 2.4	31	21.2 ± 1.6	214	23.5 ± 2.6	54	21.4 ± 2
14.14.9	11	21.3 ± 1.9	42	22.3 ± 2.5	265	24.2 ± 2.5	43	21.7 ± 1.9
**Males**								
12-12.9	36	18.4 ± 1.3	57	18.9 ± 1.3	172	22.5 ± 2.8	63	19.5 ± 2.4
13-13.9	37	18.8 ± 1.2	45	19.5 ± 1.6	221	23.3 ± 2.7	55	20.6 ± 1.7
14-14.9	58	19.9 ± 1.7	34	20.9 ± 1.8	221	23.9 ± 2.5	50	21.6 ± 2.4

Table S6.5. Triceps skinfold (mm) by age and sex for high-altitude community samples (with date of data collection)

	Ancoraimes 1977		La Paz 1983		Marquiri 1998	
	n	Median	n	Median	n	x̄ ± SD
**Females**						
11-11.9	17	7.0	16	10.5	21	10.0 ± 2.8
12-12.9	13	8.5	41	11.5	23	10.9 ± 4.1
13-13.9	14	9.0	31	13.8	24	9.7 ± 3.7
14.14.9	11	11.0	42	14.8	11	13.5 ± 4.5
**Males**						
12-12.9	36	6.0	57	8.8	15	7.2 ± 1.6
13-13.9	37	6.0	45	9.5	15	8.3 ± 2.2
14-14.9	58	6.0	34	8.8	23	7.1 ± 1.8

S7. Secular Trend Analyses

Below is a replica of Table 8 from the main text. Below the table is a line by line explanation of the table and the analyses.

Table 8

	Secular trend (z-score^W/decade)	Height-for-age (z-score^W)
1: Ancoraimes-to-Marquiri **rural trend line**: slope (z-score^W/decade)	0.340	
2: La Paz-to-El Alto **urban trend line**: slope (z-score^W/decade)	0.427	
**3: Mean secular trend**: slope (z-score^W/decade)	0.383	
4: Urbanized Locality Effect (a): urban trend line value in 1977 − Ancoraimes		0.617
5: Urbanized Locality Effect (b): La Paz − rural trend line value in 1983		0.670
6: Urbanized Locality Effect (c): urban trend line value in 1998 − Marquiri		0.801
7: Urbanized Locality Effect (d): El Alto – Ancor.-to-Marq. rural trend line in 2003		0.844
**8: Mean Urbanized Locality Effect (1977-2003)**		0.731
9: Total increase in mean height from Ancoraimes to El Alto		1.727
10: **Contribution of mean secular trend** (= 0.383*2.6 decades)		0.996 (58% of total increase)
11: **Contribution of urbanized locality effect** (= 1.727−0.996)		0.731 (42% of total increase)
12: Contribution of rural secular trend (=0.340*2.6 decades)		0.883 (51% of total increase)
13: Urbanized locality effect over and above rural secular trend (=1.727-0.883)		0.844 (49% of total increase)
14: Contribution of urban secular trend (=0.427*2.6 decades)		1.110 (64% of total increase)
15: Urbanized locality effect over and above urban secular trend (=1.727-1.110)		0.617 (36% of total increase)
16: MESA - La Paz-to-El Alto urban trend line		0.069

Line 1: This is an estimate of the average rate of improvement ("secular trend") in adolescents' height-for-age (i.e., the average rate of change in z-score^W per decade) between the Ancoraimes (1977) and Marquiri (1998) rural samples. (In all cases we take the mean of the female and male z-score^W for a sample.) In other words, this is the slope of the trend line connecting the Ancoraimes and Marquiri samples in figure 1.

Line 2: This is a similar estimate of the rate of change in adolescents' height-for-age between the La Paz (1983) and El Alto (2003) urbanized samples, i.e., this is the slope of the trend line connecting the La Paz and El Alto samples in figure 1.

Line 3:This is an estimate of the overall regional average rate of improvement ("secular trend") in adolescents' height-for-age z-score^{W}, computed as the average of lines 1 and 2. In other words, this is the average of the slopes of the two trend lines in figure 1.

Line 4:This is an estimate of the difference in adolescents' height-for-age between rural and urbanized regions at the time of the Ancoraimes sample, computed as the difference between (a) the Ancoraimes sample height-for-age, and (b) the urban height-for-age trend line (the line connecting the La Paz and El Alto samples in figure 1) at the time of the Ancoraimes sample (1977).

Line 5:This is an estimate of the difference in adolescents' height-for-age between rural and urbanized regions at the time of the La Paz sample, computed as the difference between (a) the rural height-for-age trend line (the line connecting the Ancoraimes and Marquiri samples in figure 1) at the time of the La Paz sample (1983), and (b) the La Paz sample height-for-age.

Line 6:This is an estimate of the difference in adolescents' height-for-age between rural and urbanized regions at the time of the Marquiri sample, computed analogously to line 4.

Line 7:This is an estimate of the difference in adolescents' height-for-age between rural and urbanized regions at the time of the El Alto sample, computed analogously to line 5.

Line 8:This is the average difference in adolescents' height-for-age between rural and urbanized regions at the same time (i.e., the average difference in z-score^{W} between the rural and urban trend lines in figure 1) over the period from the Ancoraimes sample (1977) to the El Alto sample (2003).

Line 9:This is the difference in adolescents' height-for-age between the Ancoraimes sample (1977, rural) and the El Alto sample (1983, urbanized).

Line 10:This is an estimate of how much of the line 9 height-for-age difference can be accounted for by the overall regional secular trend in height-for-age, computed by multiplying the line 3 slope by the time interval from the Ancoraimes sample to the El Alto sample (from 1977 to 2003 is 26 years = 2.6 decades). The percentage gives this product as a fraction of line 9.

Line 11:This is an estimate of how much of the line 9 height-for-age difference can be accounted for by the rural-to-urbanized locality effect, computed by subtracting line 10 from line 9. (Mathematically, this difference necessarily equals line 8.) The percentage gives this difference as a fraction of line 9.

Line 12:This is an estimate similar to that of line 10, but now considering the rural secular trend in height-for-age, i.e., using the line 1 slope instead of the line 3 slope.

Line 13:This is an estimate similar to that of line 11, but again considering the rural secular trend in height-for-age, i.e., subtracting line 12 from line 9.

Line 14:This is an estimate similar to that of line 10, but now considering the urbanized secular trend in height-for-age, i.e., using the line 2 slope instead of the line 3 slope.

Line 15:This is an estimate similar to that of line 11, but again considering the urbanized secular trend in height-for-age, i.e., subtracting line 14 from line 9.

Line 16:This is an estimate of the difference in adolescents' height-for-age between (a) the urbanized trend line in figure 1 at the time of the MESA sample (2006), and (b) the MESA sample.

REFERENCES CITED

1. UNESCO. Peri-Urban Landscapes; Water, Food and Environmental Security (https://en.unesco.org/events/peri-urban-landscapes-water-food-and-environmental-security). Sydney, Australia, 2014.

2. Iaquinta D, Drescher AW. Defining the peri-urban: Rural-urban linkages and institutional connections. *Land Reform, Land Settlement and Cooperatives* 2000:8-27 (https://www.researchgate.net/profile/David-Iaquinta/publication/287613842_Defining_the_peri-urban_Rural-urban_linkages_and_institutional_connections/links/588792d1aca272b7b4525792/Defining-the-peri-urban-Rural-urban-linkages-and-institutional-connections.pdf).

3. Arbona JM, Kohl B. La Paz–El Alto. *Cities* 2004;**21**:255–65.

4. Burman A. *Descolonización Aymara: Ritualidad y Política (2006-2010).* La Paz, Bolivia: Plural Editores, 2011.

5. Diaz MP. La cara aymara de la ciudad de El Alto: entre la producción social del hábitat y el mercado (1985-2012). *XI Jornadas de Sociología Facultad de Ciencias Sociales, Universidad de Buenos Aires, Buenos Aires, 2015* 2015:22.

6. Francou B, Ramirez E, Cáceres B *et al.* Glacier Evolution in the Tropical Andes during the Last Decades of the 20th Century: Chacaltaya, Bolivia, and Antizana, Ecuador. *Ambio* 2000;**29**:416–22.

7. Khlebnikova EI. High-Altitude Climate Zones and Climate Types. In: Vadimovich Gruza G (ed.). *Environmental Structure and Function: Climate System*. EOLSS, 2009.

8. de Meer K, Bergman R, Kusner JS *et al.* Differences in physical growth of Aymara and Quechua children living at high altitude in Peru. *American Journal of Physical Anthropology* 1993;**90**:59–75.

9. Mariscal CB, Tassi N, Miranda AR *et al.* eds. *Rural Migration in Bolivia: The Impact of Climate Change, Economic Crisis and State Policy*. London: Internat. Inst. for Environment and Development, 2011.

10. O'Hare G, Rivas S. Changing poverty distribution in Bolivia: The role of rural-urban migration and urban services. *GeoJournal* 2007;**68**:307–26.

11. Foster V, Irusta O. *Does Infrastructure Reform Work for the Poor? A Case Study on the Cities of La Paz and El Alto in Bolivia*. The World Bank, 2003.

12. Anderson LE, Jemio LC. *Decentralization and Poverty Reduction in Bolivia: Challenges and Opportunities*. La Paz, 2016.

13. Albó X, Galindo F. *Interculturalidad en el desarrollo rural sostenible: el caso de Bolivia: pistas conceptuales y metodológicas (https://www.bivica.org/files/interculturalidad-desarrollo-rural.pdf)*. La Paz, Bolivia: CIPCA Centro de Investigación y Promoción del Campesinado, 2012.

14. Singh EI, Vitzthum VJ. Gender differences in physical activity patterns and the effect of time spent in school on total daily energy expenditure in Bolivian children. *American Journal of Human Biology*. Vol 24. Wiley, 2012, 242.

15. Popkin BM, Adair LS, Ng SW. Global nutrition transition and the pandemic of obesity in developing countries. *Nutrition reviews* 2012;**70**:3–21.

16. Greksa LP, Spielvogel H, Paredes-Fernandez L *et al.* The physical growth of urban children at high altitude. *American Journal of Physical Anthropology* 1984;**65**:315–22.

17. Leonard WR, Leatherman TL, Carey JW *et al.* Contributions of nutrition versus hypoxia to growth in rural Andean populations. *Am J Hum Biol* 1990;**2**:613–26.

18. Shimabuku RL, Delgado CA, Nakachi G *et al.* Double Burden of Excess Weight and Anemia in Latin American Children up to 2019. *Tohoku J Exp Med* 2020;**252**:159–68.

19. Teran G, Cuna W, Brañez F *et al.* Differences in nutritional and health status in school children from the highlands and lowlands of Bolivia. *American Journal of Tropical Medicine and Hygiene* 2018;**98**:326–33.

20. Jones AD, Hoey L, Blesh J *et al.* Peri-Urban, but Not Urban, Residence in Bolivia Is Associated with Higher Odds of Co-Occurrence of Overweight and Anemia among Young Children, and of Households with an Overweight Woman and Stunted Child. *Journal of Nutrition* 2018;**148**:632–42.

21. Pérez-Cueto A, Almanza M, Kolsteren PW. Female gender and wealth are associated to overweight among adolescents in La Paz, Bolivia. *European Journal of Clinical Nutrition* 2005;**59**:82–7.

22. Pawson IG, Huicho L, Muro M *et al.* Growth of children in two economically diverse Peruvian high-altitude communities. *American Journal of Human Biology* 2001;**13**:323–40.

23. Pawson IG, Huicho L. Persistence of growth stunting in a Peruvian high altitude community, 1964–1999. *American Journal of Human Biology* 2010;**22**:367–74.

24. Hoke MK, Leatherman TL. Secular trends in growth in the high-altitude district of Nuñoa, Peru 1964-2015. *Am J Phys Anthropol* 2019;**168**:200–8.

25. Rothhammer F, Silva C. Peopling of Andean South America. *American Journal of Physical Anthropology* 1989;**78**:403–10.

26. Rothhammer F, Llop E, Carvallo P *et al.* Origin and Evolutionary Relationships of Native Andean Populations. *High Altitude Medicine & Biology* 2001;**2**:227–33.

27. Gayà-Vidal M, Moral P, Saenz-Ruales N *et al.* mtDNA and Y-chromosome diversity in Aymaras and Quechuas from Bolivia: Different stories and special genetic traits of the Andean Altiplano populations. *American Journal of Physical Anthropology* 2011;**145**:215–30.

28. Fehren-Schmitz L, Harkins KM, Llamas B. A paleogenetic perspective on the early population history of the high altitude Andes. *Quaternary International* 2017;**461**:25–33.

29. Rothhammer F, Spielman R. Anthropometric variation in the Aymara: Genetic, geographic and topographic contributions. *American Journal of Human Genetics* 1972;**24**:371–80.

30. Goedde H, Rothhammer F, Benkmann H *et al.* Genetic studies in Atacameño Indians: Serum protein and red cell enzyme polymorphisms. *Annals of Human Biology* 1985:252–9.

31. Stinson S. Variation in body size and shape among South American Indians. *Am J Hum Biol* 1990;**2**:37–51.

32. Stinson S. Child growth and the economic value of children in rural Bolivia. *Human Ecology* 1980;**8**:89–103.

33. Baya Botti A, Pérez-Cueto FJA, Monllor PAV *et al.* Anthropometry of height, weight, arm, wrist, abdominal circumference and body mass index, for Bolivian Adolescents 12 to 18 years - Bolivian adolescent percentile values from the MESA study. *Nutricion Hospitalaria* 2009;**24**:304–11.

34. Cossio-Bolaños MA, Sanchez-Macedo L, Lee Andruske C *et al.* Physical growth and body adiposity patterns in children and adolescents at high altitudes in Peru: Proposed percentiles for assessment. *Am J Hum Biol* 2020, DOI: 10.1002/ajhb.23398.
